# Non-Enzymatic MGO-Glycation of SRSF2 Drives RNA Mis-Splicing

**DOI:** 10.1021/jacs.5c20726

**Published:** 2026-05-11

**Authors:** Yang Xiao, Abdul-Vehab Dozic, Rachel Deplus, Salima Benbarche, Robert Stanley, François Fuks, Omar Abdel-Wahab, Caleb Lareau, Yael David

**Affiliations:** † 5803 Chemical Biology Program, Memorial Sloan Kettering Cancer Center, New York, New York 10021, United States; ‡ Tri-Institutional PhD Program in Chemical Biology, New York, New York 10021, United States; § 658552 Computational and Systems Biology Program, Memorial Sloan Kettering Cancer Center, New York, New York 10021, United States; ∥ Department of Physiology, Biophysics and Systems Biology, Weill Cornell Medicine, New York, New York 10021, United States; ⊥ Laboratory of Cancer Epigenetics, Faculty of Medicine, ULB-Cancer Research Center (U-CRC), 26659 Universite ´ Libre de Bruxelles (ULB), Institut Jules Bordet, Brussels 1070, Belgium; # Molecular Pharmacology Program, Sloan Kettering Institute, Memorial Sloan Kettering Cancer Center, New York, New York 10021, United States; ¶ Department of Pharmacology, Weill Cornell Medicine, New York, New York 10021, United States

## Abstract

Methylglyoxal (MGO) is a reactive metabolic byproduct of glycolysis shown to accumulate in highly glycolytic cells such as cancer cells. MGO reacts with proteins to form covalent adducts in a process termed glycation, and MGO-glycation has been linked to oxidative stress, diabetes, cancer, neurodegenerative diseases, and inflammation. Although several protein targets of MGO-glycation and their link to disease have been reported, we lack a complete understanding of MGO-glycation targets that may underlie disease progression. Here, we take a quantitative chemoproteomic profiling approach with an alkyne-functionalized MGO probe (AlkMG) to map the proteome-wide targets of MGO. A total of 494 proteins were found to be glycated under these conditions, many of which are involved in RNA processing pathways. Focusing on the serine/arginine-rich splicing factor 2 (SRSF2), we determined the sites and characterized the role that MGO-derived modifications have on its function. Biophysical modeling of site-specific glycation of SRSF2 identified residues that destabilize the native protein-RNA complex upon glycation, which we corroborated experimentally by RNA pulldown. Importantly, we found that these glycation events attenuate SRSF2’s RNA binding and alter RNA splicing, phenocopying a recurrent leukemia oncogenic SRSF2 mutation, P95H. Collectively, our study resolves glycation as a bona fide, site-specific regulatory PTM for a splicing factor and provides the first evidence for MGO-mediated mis-splicing in living cells, suggesting a new mechanistic link between MGO-glycation and disease.

## Graphical abstract

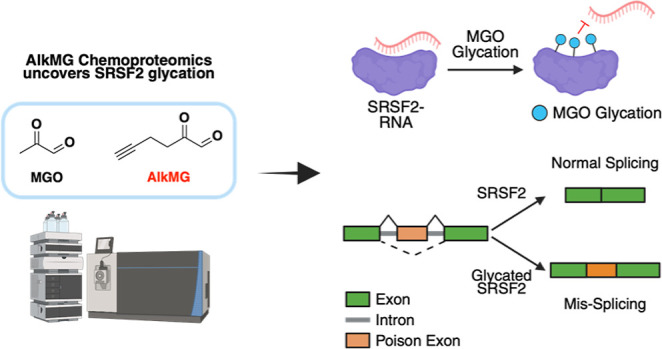

## Introduction

Metabolites, either endogenously generated or taken up from the environment, impact essential cellular functions such as energy production and signal transduction.^1^ In addition to their well-characterized roles in driving cellular metabolism, these small molecules can directly modulate the activity of proteins by acting as cofactors for enzymatic reactions and by directly forming post-translational modifications (PTMs) on amino acids side chains.^2,3^ One such metabolite is methylglyoxal (MGO), a reactive dicarbonyl which is primarily generated from glucose in the glycolysis pathway. MGO accumulates in metabolically perturbed cells that rely heavily on glycolysis, such as cancer cells. Under these conditions, elevated levels of glycolytic intermediates glyceraldehyde-3-phosphate (GA3P) and dihydroxyacetone phosphate (DHAP) spontaneously decompose to form MGO.^4,5^ Due to its ubiquitous and reactive nature, MGO is known to nonenzymatically modify nucleophilic residues on proteins, which rearrange to form a myriad of advanced glycation end-products (AGEs) adducts.^6^ These adducts accumulate over time and have been strongly linked to metabolic disorders like diabetes and to aging.^4^ Several recent studies have unveiled functional roles of MGO, through modifying key protein targets, in epigenetic regulation,^7,8^ tumorigenesis,^9,10^ and antioxidant response,^11^ highlighting the biological importance of this sugar metabolite in cells. However, due to limitations related to tracking diverse MGO adducts and enriching them, knowledge of the broad scope of proteins that are modified by MGO, and the downstream functional consequences, remain elusive. Thus, profiling MGO-modified proteins in cells will advance our discovery of new mechanistic links between protein glycation and disease.

We and others have reported the synthesis of an alkyne-functionalized MGO analogue (AlkMG) that allowed the tracking and enrichment of glycated proteins.^12,13^ We have demonstratedthat AlkMG exhibits similar reactivity to MGO *in vitro* and *in cellulo* and is detoxified through the same enzymatic pathways.^12^ Consistent with this, previous work showed that AlkMG labeling can be competed off by excess MGO in cell lysates, although relatively high concentrations of MGO are required, likely due to the abundance of potential glycation sites and the reversible nature of early glycation adducts.^13^ Importantly, we showed that AlkMG can be used to label proteins in live cells in a dose-dependent manner.^12^ Others have demonstrated the use of AlkMG in profiling MGO-modified proteins in blood and plasma.^14^

Here, we have successfully applied AlkMG in a quantitative chemoproteomic platform to map glycation targets proteome-wide. A total of 494 targets were identified to be modified in living cells, with many of them involved in RNA-processing pathways including splicing. We focused on the serine/arginine-rich splicing factor 2 (SRSF2) for further biochemical and functional characterization, because its mutation and dysregulated PTMs are known to cause pathogenic splicing in leukemia.^15–17^ Our results demonstrated that SRSF2 indeed undergoes glycation by MGO on several arginine residues specifically in the RNA recognition motif (RRM) domain. Importantly, by combining structure-based biophysical computational modeling, biochemical, and cellular assays, we showed that these glycations on SRSF2 attenuate its RNA binding and promote oncogenic mis-splicing events, suggesting that this newly identified modification may play a role in regulating splicing in cells.

## Results and Discussion

### In Situ AlkMG Chemoproteomic Profiling Reveals Multiple RNA-Processing Proteins as Targets of Glycation

To identify targets of MGO-glycation proteome-wide, we utilized AlkMG as a chemical probe to enrich for glycated substrates in cells (Figure 1A). First, we confirmed efficient protein labeling with AlkMG in HEK293T cells. HEK293T cells were treated with increasing concentrations of freshly deprotected AlkMG for 5 hours at 37 °C. After cell lysis, equal amounts of lysates were subjected to copper-catalyzed azide–alkyne cycloaddition (CuAAC) with Biotin-PEG3-Azide to label AlkMG-modified proteins. Biotinylated proteins were separated on an SDS-PAGE and AlkMG-modified species were visualized using a streptavidin-IRDye800 antibody. As expected, AlkMG treatment resulted in a dose-dependent increase in streptavidin signal, indicating labeling of multiple cellular proteins (Figure 1B). These AlkMG-labeled proteins were subsequently identified via an unbiased quantitative chemoproteomics workflow. In brief, HEK293T cells were treated with either PBS, 1.5 mM MGO or 1.5 mM AlkMG for 5 hours at 37 °C. Following a click reaction, labeled proteins (Figure S1) were enriched by streptavidin agarose and subjected to proteomics preparation for Tandem Mass Tag-based liquid chromatography coupled to mass-spectrometry (TMT-LC-MS/MS) (full proteomics data is available as Data set S1). As a control for the proteomics analysis, we compared the AlkMG-treated with MGO-treated cells, which account for dicarbonyl stress-induced global protein abundance, from three independent experiments. This analysis yielded a total of 494 positive hits, determined by fold-change ≥2.0 and *p*-value ≤0.05 (Figure 1C). The veracity of these proteomics data was validated by immunoblotting of several enriched proteins, including SRSF2, serine/arginine-rich splicing factor 1 (SRSF1), and high-mobility group protein A1 (HMGA1), as well as MCM2, which was not identified as a hit, used as the negative control (Figure 1D).

**Figure 1:**
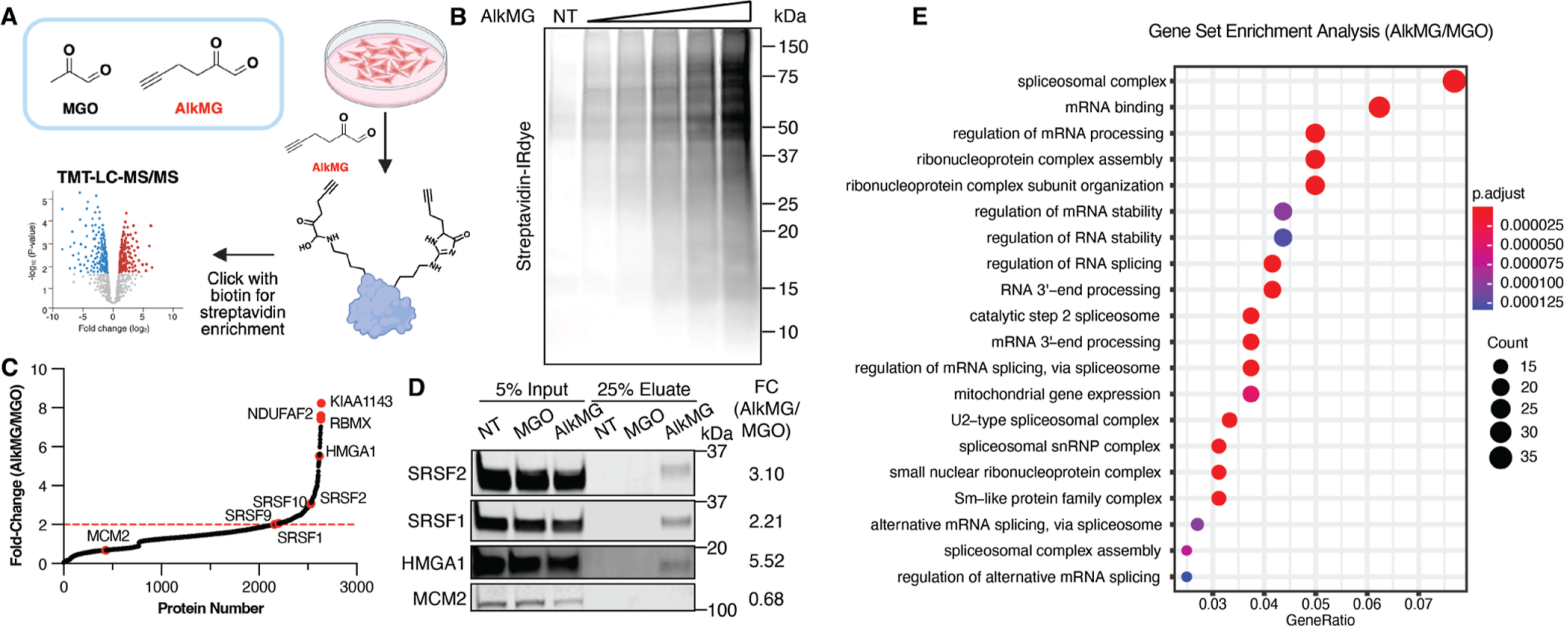
AlkMG chemoproteomic profiling in HEK293T cells revealed multiple RNA-processing proteins as glycation targets. (A) Quantitative TMT-LC-MS/MS AlkMG chemoproteomics workflow. (B) HEK293T cells were treated with PBS (NT) or increasing concentrations of AlkMG (0.1–1.5 mM) for 5 hours, followed by lysis, Biotin-PEG3-Azide click labeling, and immunoblotting analysis with IRDye 800CW Streptavidin. (C) Plot of fold change in TMT-LC-MS/MS quantification for 1.5 mM AlkMG versus 1.5 mM MGO treatment in HEK293T cells. Proteins above the dashed red line (fold-change ≥2.0 and p-value ≤0.05) are designated positive hits. (D) Immunoblotting analysis confirming several enriched targets from the AlkMG chemoproteomics. MCM2 was used as a negative control. Fold-change (FC) values of the corresponding proteins are listed. (E) Gene Set Enrichment Analysis of all proteins identified in the AlkMG-treated HEK293T cells compared to the MGO-treated HEK293T cells.

Subsequent Gene Set Enrichment Analysis (GSEA) analysis of the chemoproteomics data revealed that proteins involved in RNA binding and RNA processing are significantly enriched (Figure 1E), suggesting that RNA-related processes may be targets of MGO-glycation in living cells. Interestingly, proteins that are part of the spliceosome complex were the most significantly represented (Figure 1E), which prompted the question of whether glycation of these proteins can affect splicing in cells.

### Validation of SRSF2 as a Target of MGO-Glycation

To evaluate the impact of MGO-glycation on RNA splicing, we chose to follow-up on SRSF2, which is a key splicing factor which mutation was recently shown to drive leukemia.^16,18^ As a splicing factor, SRSF2 binds exonic splicing enhancer (ESE) motifs and facilitates both constitutive and alternative splicing.^16,19^ It consists of two major domains: an N-terminal RRM that mediates sequence-specific binding to pre-mRNA, and a C-terminal arginine/serine-rich (RS) domain that facilitates protein–protein interactions and spliceosome assembly.^15,20^ Importantly, PTMs such as phosphorylation and acetylation were identified to exist on SRSF2 and alter its stability, subcellular localization, and RNA-binding specificity, thus regulating its splicing activities.^17,21,22^ However, there is currently no known nonenzymatic covalent modification (NECM) identified to occur on SRSF2. To confirm MGO-glycation indeed accumulates on SRSF2, we purified its RRM domain (AA 1–101)^15^ (Figure S2) and performed an *in vitro* glycation assay with MGO. We purified the RRM domain because the intrinsically disordered RS domain of the full-length SRSF2 hampers soluble expression and purification, whereas the RRM domain itself is stably folded and can be readily purified.^15,23^ Immunoblotting with a commercially available pan-MGO antibody confirmed the formation of MGO adducts on SRSF2 RRM domain, as well as higher molecular weight cross-linked species, consistent with the well-established propensity of MGO to induce intermolecular cross-linking,^7,11^ indicating that SRSF2 is subjected to MGO-glycation *in vitro* (Figure 2A). To further verify MGO-glycation of SRSF2 in a more physiologically relevant system we overexpressed Flag-tagged full-length SRSF2 in HEK293T cells and treated them with increasing concentrations of MGO that mimic long-term exposure to MGO in disease states.^7,10,11^ Immunoprecipitation with anti-Flag beads (Flag-IP) confirmed the dose-dependent glycation of full-length SRSF2 (Figure 2B). Using the same workflow on truncated SRSF2 mutants, we confirmed that both N-terminal and C-terminal domains of SRSF2 are glycated in live cells, albeit both to a lesser extent compared with the full-length (Figure S3), possibly due to the lower stability and more rapid turnover of the truncated proteins. Together, our *in vitro* and *in cellulo* assays confirmed SRSF2 as a target of MGO-glycation.

**Figure 2:**
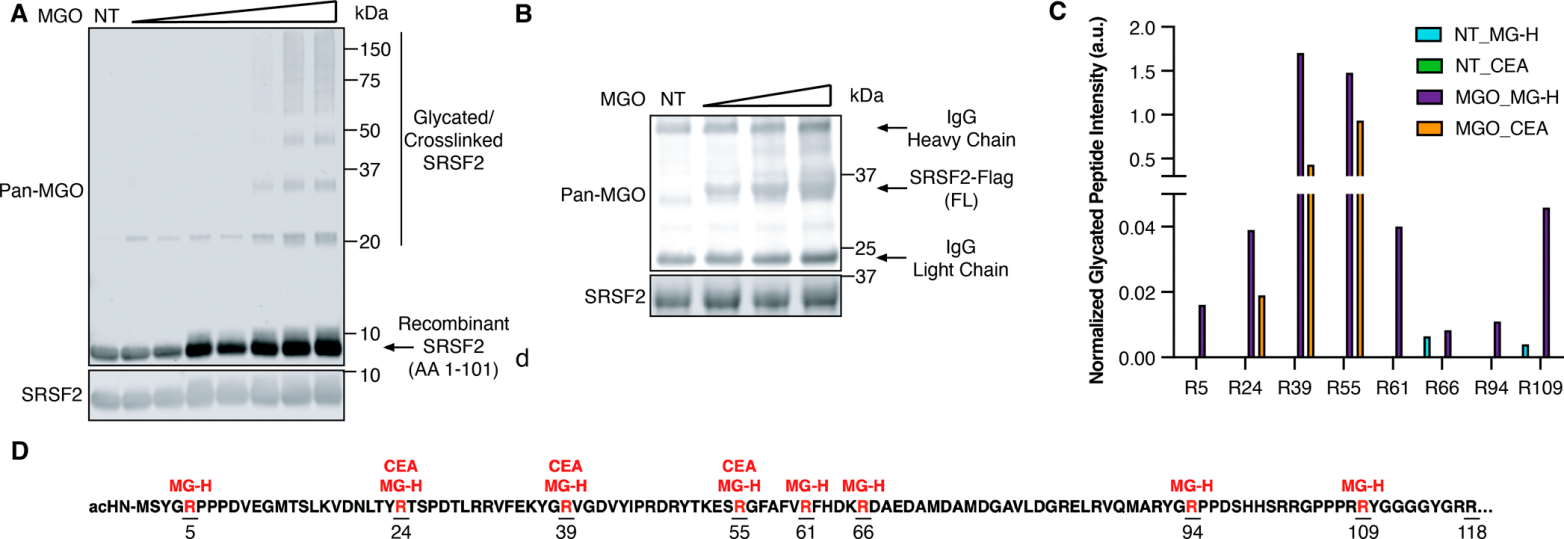
SRSF2 is a bona fide target of MGO-glycation. (A) *In vitro* glycation assay of recombinant SRSF2 RNA recognition motif (RRM) domain (AA 1–101). SRSF2 was treated with either PBS (NT) or increasing concentrations of MGO (0.1–20 mM) for 2 hours at 37 °C, followed by immunoblotting analysis with a pan-MGO antibody. (B) Glycation of SRSF2 in live cells. HEK293T cells were transfected with a full-length Flag-tagged SRSF2 and treated with PBS (NT) or increasing concentrations of MGO (0.5–1.5 mM) for 4 hours, followed by lysis, Flag-IP, and immunoblotting analysis with a pan-MGO antibody. (C) Relative intensities of glycated peptides at individual arginine sites on SRSF2, determined by IP-LC-MS/MS from 0.5 mM MGO-treated HEK293T cells overexpressing Flag-tagged SRSF2. Peptide intensity was normalized to the corresponding unmodified peptide. (D) Schematic representation of glycation sites on SRSF2.

To determine which arginine residues of SRSF2 are directly modified by MGO in live cells, we optimized a protease digestion protocol and used liquid chromatography-tandem mass spectrometry (LC–MS/MS) to map the glycation sites. We used a similar Flag-IP assay to enrich glycated full-length SRSF2 from HEK293T cells that were untreated or treated with MGO. By targeting previously reported stable MGO-glycation adducts,^6^ we identified several arginines on the RRM to be modified by MGO, forming either methylglyoxal-derived hydroimidazolone (MG-H, [M+54.01]) or carboxyethyl-arginine (CEA, [M+72.02) or both (Figures 2C,D and S4, full proteomics data available as Data set S2). Notably, three out of the four arginines (R5, R61, R91, and R94) known to directly interact with RNA in the nucleotide binding pocket^24^ were also identified to be glycated. Although the C-terminal RS domain contains multiple arginines and is therefore predicted to be highly susceptible to glycation, we were unable to detect peptides from this region (Figure S5) due to its amino acid composition and extensive post-transitional modifications, which collectively hinder proteolytic efficiency, ionization, and confident peptide assignment. This limitation is consistent with prior proteomic studies of SR proteins.^25^ However, we focus on SRSF2’s RNA binding properties because as we demonstrate below, glycation on SRSF2 specifically alters the function of its RRM domain without affecting the RS domain.

### Glycation Attenuates SRSF2’s RNA Binding Capability but Not Its Interaction with Other Spliceosome Factors

The activity of SRSF2 is highly regulated by extensive and reversible phosphorylation and acetylation.^17,21^ Since we and others have previously shown that glycation is a functional PTM on many cellular proteins,^7,9–11,26^ we hypothesized that glycation on SRSF2 affects its functions. To test this, we used a co-immunoprecipitation (Co-IP) assay to first evaluate whether MGO-glycation impacts SRSF2’s ability to interact with other key components of the spliceosomal complex. We overexpressed Flag-tagged SRSF2 in HEK293T cells and treated those with increasing concentrations of MGO. To minimize the effects of potential glycation occurring on SRSF2’s interacting partners, we first immunoprecipitated SRSF2 from MGO-treated cells and followed with incubation with whole cell lysate generated from untreated HEK293T cells in order to enrich for its interacting partners. RNase was added during the co-immunoprecipitation step to eliminate indirect associations bridged by RNA molecules. Flag-tagged GFP enriched from untreated HEK293T cells was used as a negative control. Interestingly, two key known interactors of SRSF2—U2AF1 and snRNP70—showed similar binding to both glycated SRSF2 and unmodified SRSF2 (Figure 3A,B), suggesting that SRSF2’s protein–protein interactions through the RS domain are unaffected by MGO-glycation. We speculate that RS-domain protein–protein interactions are primarily regulated by serine phosphorylation,^27,28^ rendering them relatively insensitive to arginine glycation. This observation further supports our focus on glycation events within the RRM, as it suggests that functional disruption arises primarily from modification of RNA-contact arginines rather than impaired recruitment of splicing cofactors.

**Figure 3:**
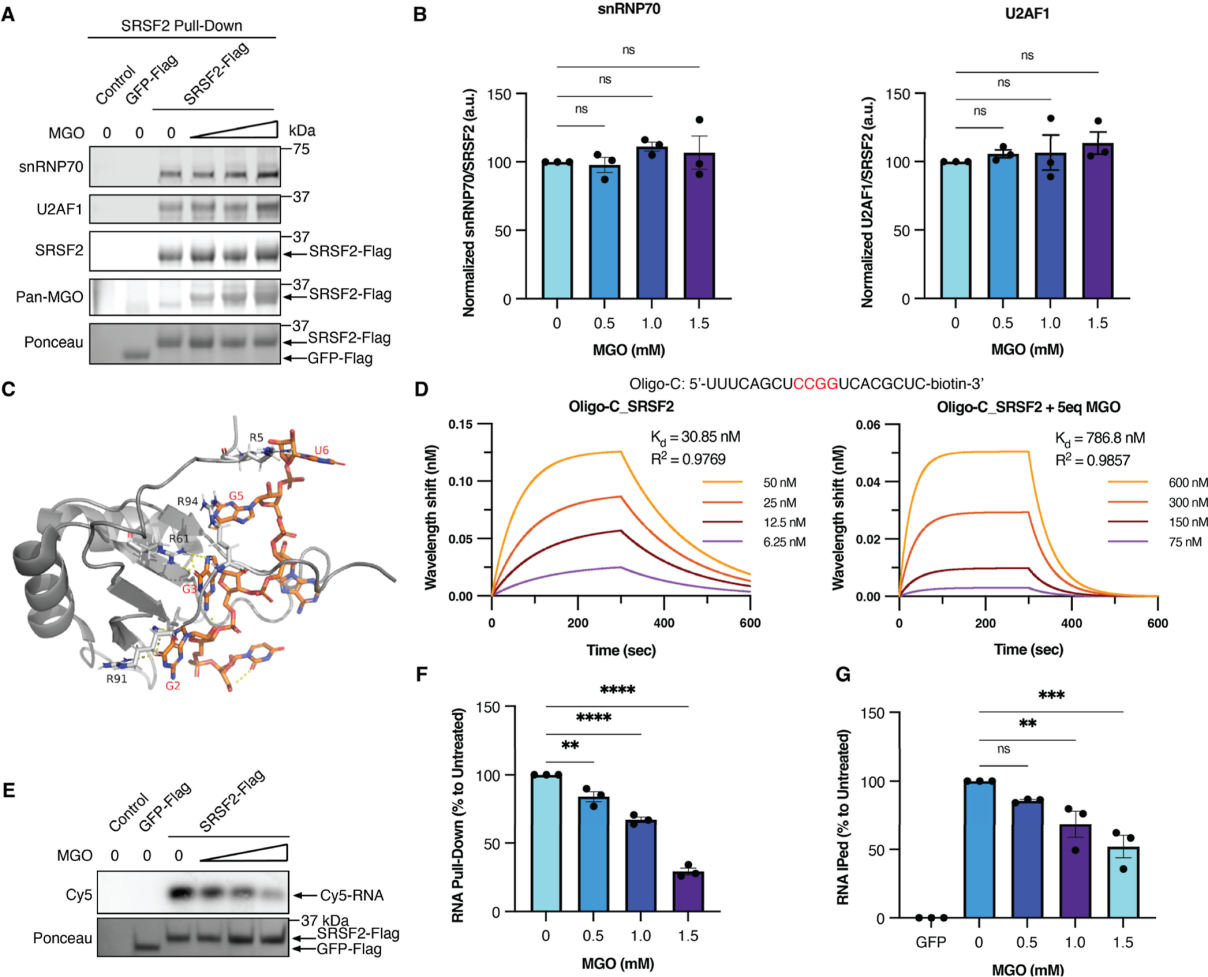
MGO-glycation attenuates SRSF2 RNA-binding affinities. (A) Flag-SRSF2 isolated from SRSF2-overexpressing HEK293T cells that were treated with increasing concentrations of MGO (0–1.5 mM) for 4 hours was used in co-immunoprecipitation (co-IP) with whole cell lysate of untreated wild type HEK293T cells generated in the presence of RNase. The levels of SRSF2 and its interacting partners were assessed by immunoblotting with anti-snRNP70 and anti-U2AF1 antibodies. (B) Quantification of the co-IP levels of snRNP70 and U2AF1 from 3 independent repeats of the experiment presented in (A) normalized to SRSF2. (C) Solution structure of human SRSF2 RRM in complex with 5′-UGGAGU-3′ (PDB: 2LEC) indicating the key arginines in Black that are targets of glycation and positions of the RNA oligo. (D) Recombinant SRSF2 RRM was treated with either PBS or 2.5 mM MGO for 1 hour at 37 °C, desalted to remove excess MGO, and then incubated with a biotinylated CCGG-containing 20-mer RNA oligo. The binding affinity was assessed by the determining the dissociation constant (*K*_d_) using BLI. (E) Full-length Flag-tagged glycated SRSF2 was isolated from HEK293T cells treated with increasing concentrations of MGO (0–1.5 mM) for 4 hours. Isolated SRSF2 was incubated with Cy5-labeled CCUG-containing 19-mer RNA oligo, after which the beads were washed and bound RNA was evaluated by Cy5 fluorescence signal. (F) Quantification of the Cy5-labeled RNA oligo pull-downed from 3 independent repeats of the experiment presented in (E) normalized to Untreated. (G) Quantification of the total RNA pull-downed by SRSF2 from 3 independent repeats of the RNA immunoprecipitation experiment presented in Figure S6 normalized to Untreated.

Based on the available co-crystal structures of SRSF2 in complex with RNA oligo (PDB: 2LEB and 2LEC),^24^ several arginines (R5, R61, R91, and R94) are directly involved in binding with the RNA transcript (Figure 3C). R5, R61, and R94 were confirmed to be modified by MGO in our LC–MS/MS analysis (Figure 2C,D). Therefore, we hypothesized that glycation of these arginines will interfere with SRSF2’s properties of RNA binding. To directly investigate the impact of SRSF2 glycation on its RNA binding affinity, we utilized biolayer interferometry (BLI) to measure the affinity of the recombinant SRSF2 RRM’s to its consensus RNA motif. We used a biotinylated CCGG-containing 20-mer RNA oligo based on previous studies,^18^ since the motif for SRSF2 is SSNG, with S = C/G and N = A/C/G/U. We found that compared with untreated SRSF2, MGO-treated SRSF2 exhibits a significantly lower affinity toward its RNA substrate with dissociation constant, *K*_d_ values, shifting from 30.85 nM to 786.8 nM (Figure 3D). These results indicate that MGO-glycation significantly disrupts SRSF2’s high-affinity recognition of SSNG motif on RNA, which is central to SRSF2’s functions.^15,29^ To orthogonally validate the BLI data, we immunoprecipitated full-length SRSF2 from HEK293T cells, which were either untreated or pretreated with MGO. The enriched SRSF2 was used in a pull-down assay with a Cy5-labeled CCUG-containing 19-mer RNA oligo. Following stringent washes, bound RNA was eluted and visualized by Cy5 fluorescence. Gratifyingly, the same glycation-induced attenuation in RNA binding was observed for the full-length SRSF2 (Figure 3E,F). To evaluate the impact of SRSF2 glycation on its overall RNA binding capacity, beyond the single motif, we performed RNA immunoprecipitation (RIP) with enriched SRSF2 from untreated or MGO-treated HEK293T cells and total RNA extracted from untreated HEK293T cells. We found that SRSF2 glycation induces a dose-dependent loss in the total amount of RNA that remained bound to SRSF2 (Figures 3G and S6), which is consistent with our other *in vitro* observations.

### Rosetta Modeling Predicts Key SRSF2 MGO-Glycation Site that Significantly Weakens its RNA Binding

Because site-specific installation of MGO adducts at individual arginines is experimentally challenging, we turned to structure-based computational modeling to predict how the mass-spectrometry identified glycated arginines may alter SRSF2-RNA interaction. Motivated by recent progress in modeling protein-nucleic acid complexes and relative binding affinity,^30,31^ we implemented a Rosetta biophysical computational modeling tailored to the SRSF2 RRM-RNA complex in order to predict the impact of glycation on the relative binding affinity. Guided by previously solved SRSF2-RNA structures (PDB: 2LEB and 2LEC),^24^ we focused on four arginine residues within the RNA-binding pocket (R5, R61, R91, and R94), three of which (R5, R61, and R94) we identified as glycated in cells by our LC–MS/MS (Figure 2C,D). MG-H1 was chosen as the modeled adduct as it is the first stable and the most abundant MGO-derived adduct.^6,32,33^ MG-H1-modified arginine was incorporated into the structure as a noncanonical amino acid by generating a full rotamer library from the “parent” arginine residue.^34^ For each arginine residue, binding free energy (Δ*G*) was computed in Rosetta energy unit (REU) using the Rosetta-Vienna RNP-ΔΔ*G* weight set, which is optimized for computing protein-RNA interactions.^35^ Each protein-RNA complex was energetically minimized 100 times where the 40 lowest–energy complexes were used to estimate Δ*G* values for the complex, protein, and RNA states (Figure 4A).

**Figure 4:**
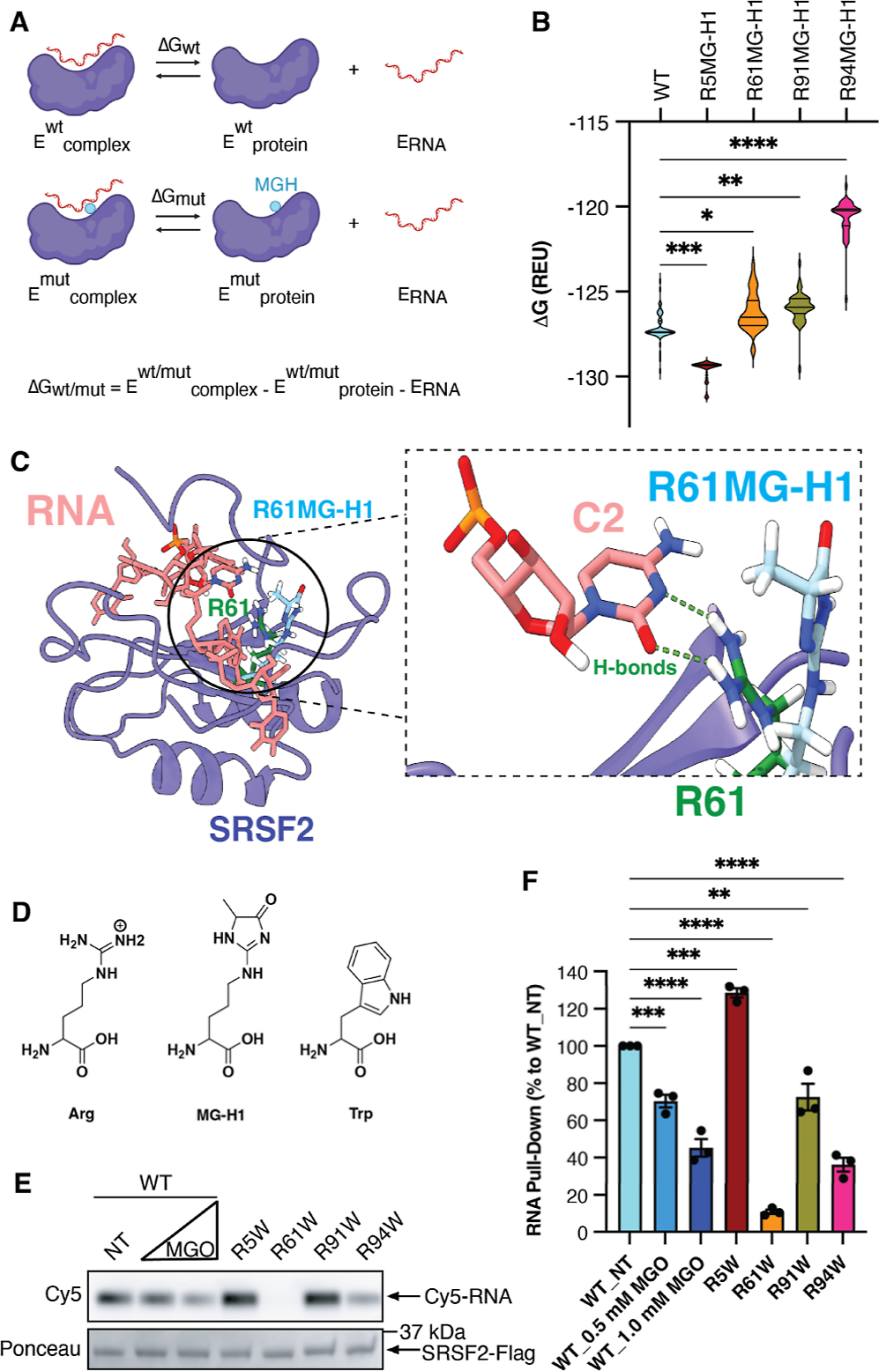
Computational modeling and experimental validation identified crucial residues for glycation-induced loss of SRSF2-RNA binding. (A) Rosetta-based SRSF2-RNA binding energetic prediction workflow. (B) The Δ*G* of WT and various MG-H1-modified site mutants SRSF2 binding to UCCUGU calculated by the Rosetta–Vienna RNP-ΔΔ*G* protocol.^35^ (C) Overlay of minimized R61 and R61MG-H1 SRSF2 complexes with UCCUGU shows a steric clash with the C2 base and disruption of hydrogen bonding upon glycation. (D) Chemical structures of arginine, MG-H1-modified arginine, and tryptophan for the purpose of chemical-biophysical mimic of MG-H1-modified arginine. (E) Full-length Flag-tagged WT SRSF2 isolated from HEK293T cells either treated or untreated with MGO (0.5/1.0 mM) and various R-to-W mutant SRSF2 isolated from untreated HEK293T cells were incubated with Cy5-labeled CCUG-containing 19-mer RNA oligo, after which the beads were washed and bound RNA was evaluated by Cy5 fluorescence signal. (F) Quantification of the Cy5-labeled RNA oligo pull-downed from 3 independent repeats of the experiment presented in (E) normalized to WT Untreated.

Consistent with our *in vitro* and *in cellulo* data demonstrating a reduced affinity of the glycated SRSF2 to its RNA substrate, our Rosetta calculations predicted that substituting arginine with MG-H1 at R61, R91, and R94 decreased the Δ*G*, indicating a weaker SRSF2-RNA binding (Figure 4B). In contrast, MG-H1 modification at R5 led to a higher Δ*G*, suggesting a stronger SRSF2-RNA binding (Figure 4B). Notably, R61MG-H1 emerged as the most destabilizing substitution. In the predicted structural models, R61MG-H1 projects into the RNA groove, sterically clashing with the cytosine at position C2 of the modeled RNA. This glycated arginine also disrupts the guanidinium-mediated hydrogen-bond network that stabilizes the native complex (Figure 4C). Similar changes were observed for MG-H1 at R91 and R94 (Figure S7). Notably, this steric hindrance mechanism resembles that proposed for the recurrent leukemia-associated SRSF2 P95H mutation, in which the bulkier histidine residue at position 95 disrupts RNA contacts through steric clash with the RNA backbone.^18^

To functionally test our Rosetta predictions, we adopted a chemical-biophysical mimicry strategy. Since MG-H1 on arginine neutralizes the guanidinium charge and adds bulky heterocyclic mass that perturbs hydrogen binding, we decided to use tryptophan to recapitulate these features (Figure 4D). We generated R-to-W mutants at the four RNA-contacting arginines (R5, R61, R91, R94) by site-directed mutagenesis, expressed these with a Flag-tag in HEK293T cells, and immunoprecipitated them for RNA-binding assay with the Cy5-labeled RNA oligo, as described above. Strikingly, the R61W mutant showed the greatest loss in RNA binding, nearly abolishing the capturing of the fluorescent RNA probe, whereas R91W and R94W displayed modest decreases (Figure 4E,F). Consistent with our Rosetta predictions, R5W led to a modest increase in RNA binding (Figure 4E,F). Overall, these data validate the computational prediction and demonstrate that R61 is the dominant glycation vulnerability in the SRSF2 RRM as it sterically invades the RNA groove and disrupts the cytosine-directed hydrogen-bond network that stabilizes the native complex (Figure 4C).

### Glycation Diminishes SRSF2 Engagement with Target Transcripts and Promotes Mis-Splicing and Degradation of EZH2 in Cells

To study SRSF2 binding to its native RNA substrates, we performed RNA immunoprecipitation-qPCR (RIP-qPCR) in HEK293T cells. To minimize the effects of glycation on transcript abundance and stability, we isolated total RNA from untreated HEK293T cells and incubated them with SRSF2 isolated from HEK293T cells either untreated or treated with MGO (Figure 5A). Because methylglyoxal can also modify nucleotides,^36,37^ this experimental design allowed us to isolate the effect of SRSF2 glycation on RNA binding while ensuring that the RNA substrates themselves were not exposed to MGO. Known SRSF2-binding transcripts (Enhancer of zeste homologue 2 (EZH2), Integrator complex subunit 3 (INTS3), and Ubinuclein 1 (UBN1)) were evaluated for their binding to SRSF2 as measured by qPCR, with 14-3-3 protein zeta/delta (YWHAZ), a housekeeping transcript not bound by SRSF2, as the negative control.^18^ Our results reveal a dose-dependent loss of SRSF2 engagement with EZH2, INTS3, and UBN1 pre-mRNAs, whereas the negative-control transcript YWHAZ showed no enrichment binding to SRSF2 (Figures 5B and S8). These data establish that glycation weakens SRSF2-RNA interactions on native targets, consistent with our biochemical pull-down and computational predictions.

**Figure 5:**
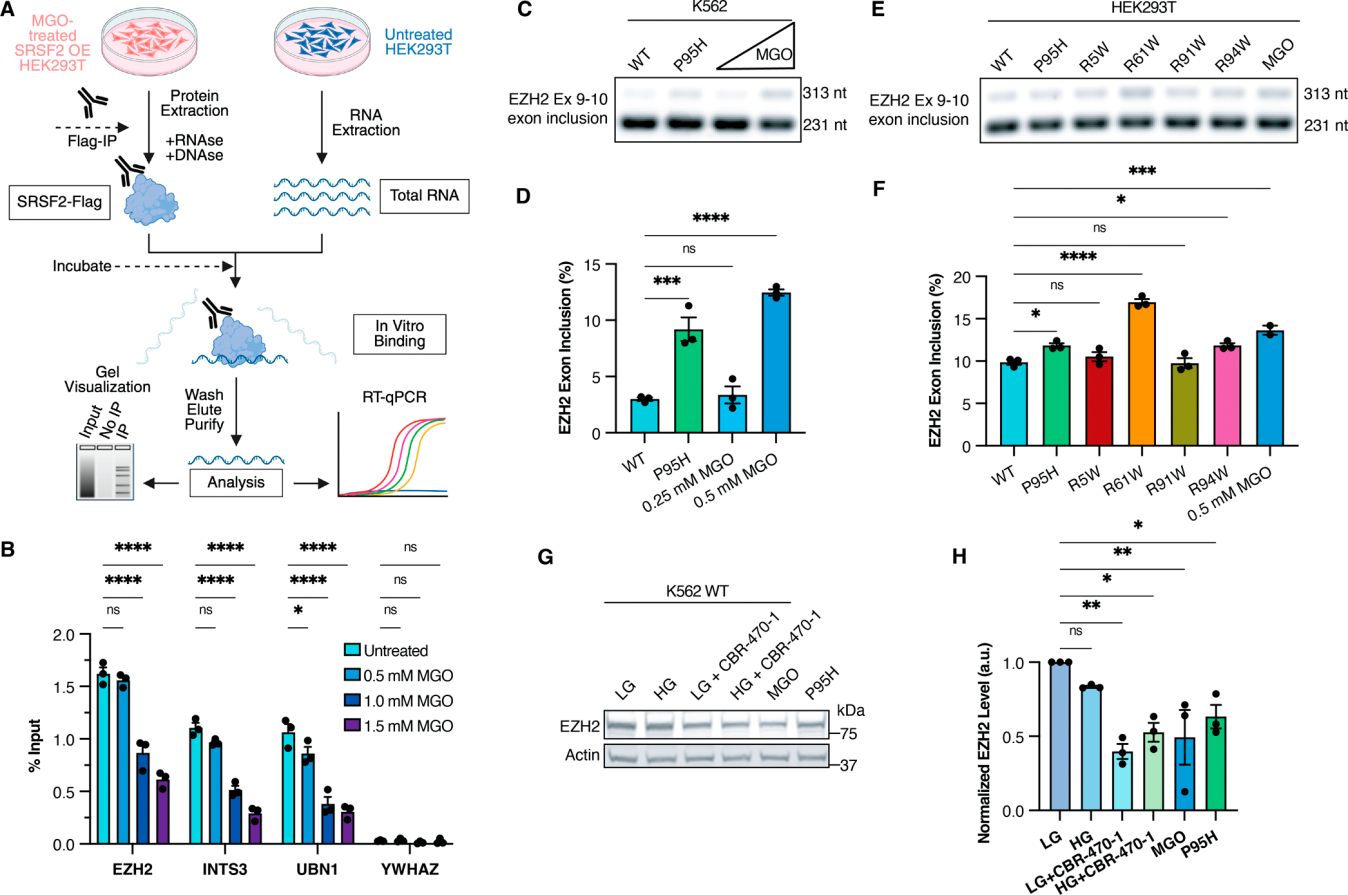
Glycation alters SRSF2’s RNA binding profile and promotes cellular mis-splicing events. (A) RNA immunoprecipitation-qPCR (RIP-qPCR) workflow used in (B). (B) Full-length Flag-tagged SRSF2 was isolated from HEK293T treated with increasing concentrations of MGO (0–1.5 mM) for 4 h, followed by lysis, Flag-IP, and RIP-qPCR analysis of known SRSF2 binding transcripts (EZH2, INTS2, UBN1) with YWHAZ as a negative control. (C) RT-PCR of an EZH2 exon inclusion event in WT SRSF2 K562 cells either treated or untreated with MGO (0.25/0.5 mM) and untreated P95H SRSF2 K562 cells. (D) Quantification of the EZH2 exon inclusion event from 3 independent repeats of the experiment presented in (C). (E) RT-PCR of an EZH2 exon inclusion event in WT SRSF2-overexpressing HEK293T cells either treated or untreated with MGO (0.5 mM) and various untreated R-to-W mutant SRSF2-overexpressing HEK293T cells. (F) Quantification of the EZH2 exon inclusion event from 3 independent repeats of the experiment presented in (F). (G) Immunoblotting analysis of EZH2 protein level in WT SRSF2 K562 cells cultured under low-glucose (LG, 5 mM) or high-glucose (HG, 25 mM) conditions, either untreated or treated with 20 μM of CBR-470-1 or 100 μM of MGO overnight. P95H SRSF2 K562 cell line was used as a positive control. (H) Quantification of EZH2 protein level from 3 independent repeats of the experiment presented in (G).

We next tested whether the decreased RNA binding of glycated SRSF2 translates to mis-splicing events in cells. It has been well-established that the oncogenic P95H mutation of SRSF2, which is highly recurrent in myeloid malignancies and functions as a key pathogenic driver, alters SRSF2’s RNA binding profile and promotes the inclusion of a highly conserved “poison” cassette exon of EZH2.^16^ This exon introduces a premature termination codon, leading to nonsense-mediated decay of EZH2 transcripts and consequent reduction of EZH2 protein levels.^16^ Therefore, we focused on the splicing event in EZH2 as a disease-relevant model of SRSF2 dysfunction, allowing us to bridge *in vitro* binding defects with cellular functional consequences. To benchmark our glycation phenotype against the established oncogenic P95H SRSF2 signature, we first assessed EZH2 splicing in K562 leukemia cells, a system previously used to define the P95H-driven inclusion of the EZH2 “poison” exon. As expected, by isoform-specific RT-PCR, K562 cells that stably expressed P95H SRSF2 had a marked increase in exon inclusion relative to WT SRSF2 (Figures 5C,D and S9). Strikingly, 0.5 mM MGO treatment phenocopied the P95H-induced exon-inclusion pattern (Figures 5C,D and S9), demonstrating that MGO-glycation can recapitulate the splicing defect of a recurrent cancer-driving mutation.

We next extended this analysis to HEK293T cells, where we could transiently express individual R-to-W mutants. In this setting, both 0.5 mM MGO treatment and R61W SRSF2 expression increased inclusion of the EZH2 “poison” exon, with the largest effect observed for R61W (Figures 5E,F and S10), consistent with our computational and biochemical data (Figure 4B,E,F). While the differences in the magnitude of exon inclusion in HEK293T cells were smaller relative to K562, likely due to transient overexpression rather than stable allelic replacement, the relative ranking of effects mirrored those observed in K562 leukemia cells. Together, these data show that site-specific disruption of the RRM, either genetically or via glycation, drives the same oncogenic EZH2 splicing program as P95H SRSF2, highlighting metabolite-driven glycation as a functional mimic of a pathogenic allele.

Finally, to identify whether the protein product of EZH2 is altered through mis-splicing by glycation on SRSF2, we performed immunoblotting analysis of K562 cell lines under various glycation-promoting conditions. Gratifyingly, MGO treatment led to a decrease in EZH2 protein level, comparable to the one observed in K562 SRSF2 P95H mutant cell line (Figure 5G,H). A similar effect was detected when endogenous MGO levels were upregulated in WT K562 cells by culturing them in 25 mM glucose (the standard high-glucose formulation used in cancer cell culture and the main source of triose-phosphate-derived MGO) or treating them with CBR-470-1 (a small-molecule inhibitor of the glycolytic enzyme PGK1 that induces the accumulation of endogenous intracellular MGO^11^) (Figure 5G,H). Together, these results link nonenzymatic glycation to a disease-relevant splicing outcome: metabolite-driven modification of an RNA-contact arginine is sufficient to attenuate SRSF2–RNA engagement, trigger EZH2 poison-exon inclusion, and reduce EZH2 protein abundance.

## Conclusions

Using proteome-wide profiling, we uncovered RNA-processing proteins to be major targets of MGO-glycation. Focusing on a key splicing factor SRSF2, we found that glycation on arginine residues in its RNA binding domain weakens its RNA binding affinity. Computational biophysical modeling identified specific residues, primarily R61, that drive the glycation-induced loss in RNA binding, which we experimentally validated. Functionally, glycation on SRSF2 alters its RNA binding profile, triggers oncogenic EZH2 mis-splicing, and reduces EZH2 protein abundance. Our study defines glycation as a bona fide, site-specific regulatory modification on a splicing factor and establishes a direct mechanistic link between metabolic stress and RNA mis-splicing. While methylglyoxal can also react with nucleic acids, the use of purified glycated protein, untreated RNA substrates, computational modeling, and glycation-mimicking mutants collectively support a direct role for SRSF2 modification in driving the observed RNA-binding and splicing phenotypes. The integrated computational-experimental framework presented here provides a generalizable strategy to dissect metabolite-derived PTMs across diverse cellular pathways.

## Supplementary Material







## Data Availability

The underlying code for the computational modeling will be available at the following link: https://github.com/clareaulab/Protein-RNA_modeling.

## Notes

Raw proteomics data set S1 for AlkMG chemoproteomics (XLSX)
Raw proteomics data set S2 for MGO glycation site mapping (XLSX)
Supplementary Figures S1–10; general materials and methods; compound synthesis and characterization (PDF)
