# Supplementary material for: Non-Enzymatic MGO-Glycation of SRSF2 Drives RNA Mis-Splicing
Source: J Am Chem Soc. 2026 May 11;148(19):19502–10. doi: 10.1021/jacs.5c20726 (PMC13195657; doi:10.1021/jacs.5c20726)
Supplement: Supplementary file 3 [file ja5c20726_si_003.pdf]

Non-Enzymatic MGO-Glycation of SRSF2 Drives RNA Mis-Splicing

Yang Xiao[1,2], Abdul-Vehab Dozic[3,4], Rachel Deplus[5], Salima Benbarche[6], Robert Stanley[6], François Fuks[5], Omar Abdel-Wahab[6], Caleb Lareau[3,4], and Yael David[1,2,7,8,*]

[1]Chemical Biology Program, Memorial Sloan Kettering Cancer Center, New York, New York, 10021, United States; [2]Tri-Institutional PhD Program in Chemical Biology, New York, New York, 10021, United States; [3]Computational and Systems Biology Program, Memorial Sloan Kettering Cancer Center, New York, New York, 10021, United States; [4]Department of Physiology, Biophysics and Systems Biology, Weill Cornell Medicine, New York, New York, 10021, United States; [5]Laboratory of Cancer Epigenetics, Faculty of Medicine, ULB-Cancer Research Center (U-CRC), Université libre de Bruxelles (ULB), Institut Jules Bordet, Brussels, 1070, Belgium; [6]Molecular Pharmacology Program, Sloan Kettering Institute, Memorial Sloan Kettering Cancer Center, New York, New York, 10021, United States; [7]Department of Physiology, Biophysics and Systems Biology, Weill Cornell Medicine, New York, New York, 10021, United States; [8]Department of Pharmacology, Weill Cornell Medicine, New York, New York, 10021, USA; *Corresponding Author.

SUPPORTING INFORMATION

Table of Contents

A. **Supplementary Figures**
B. **General Materials and Methods**
C. **Compound Synthesis and Characterization**
D. **References**

A. Supplementary Figures

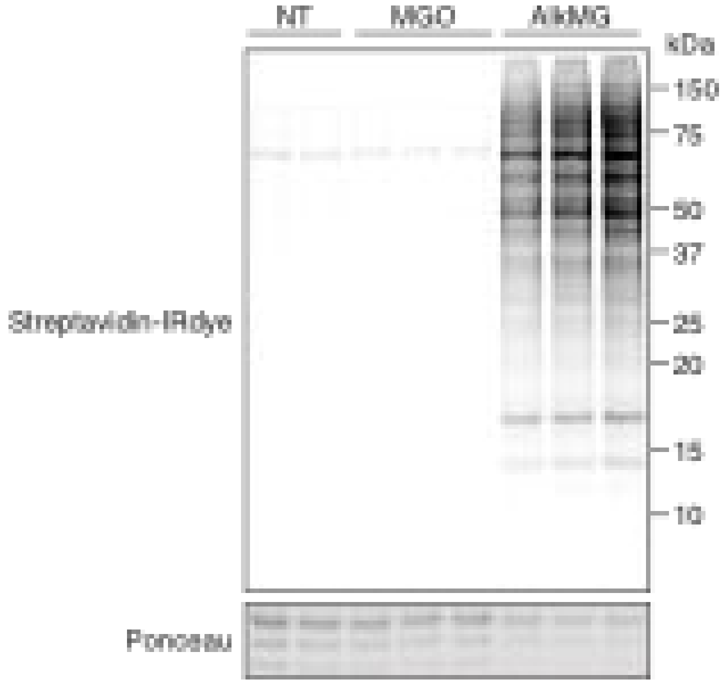

Figure S1. Labelling of cellular proteins by AlkMG. HEK293T cells were treated with PBS (NT), 1.5 mM MGO, or 1.5 mM AlkMG for 5 h, followed by lysis, Biotin-PEG3-Azide click labeling, and immunoblotting analysis with streptavidin-IRDye800.

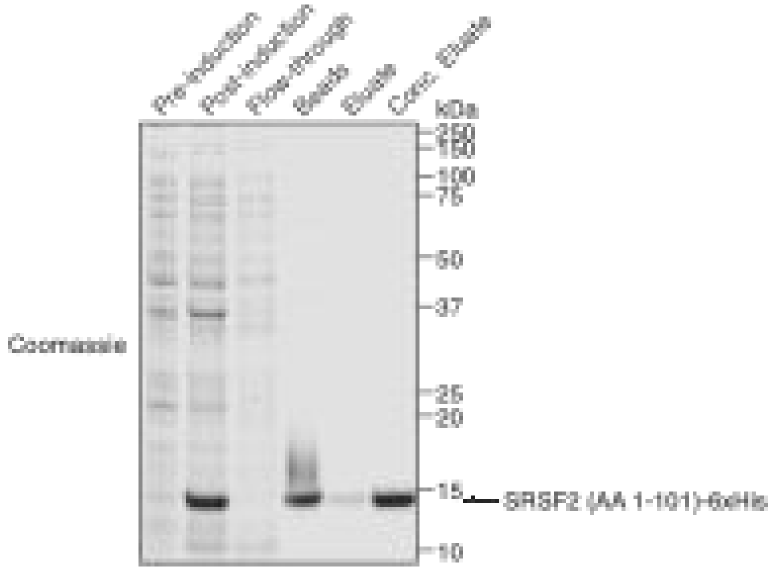

Figure S2. Expression and purification of 6xHis-tagged wild-type (WT) SRSF2 (AA 1-101). Lysates from pre- and post-induction, flow-through from Ni-NTA Resin column, Ni-NTA beads, the final eluate, and the concentrated eluate were subjected to SDS-PAGE and Coomassie staining.

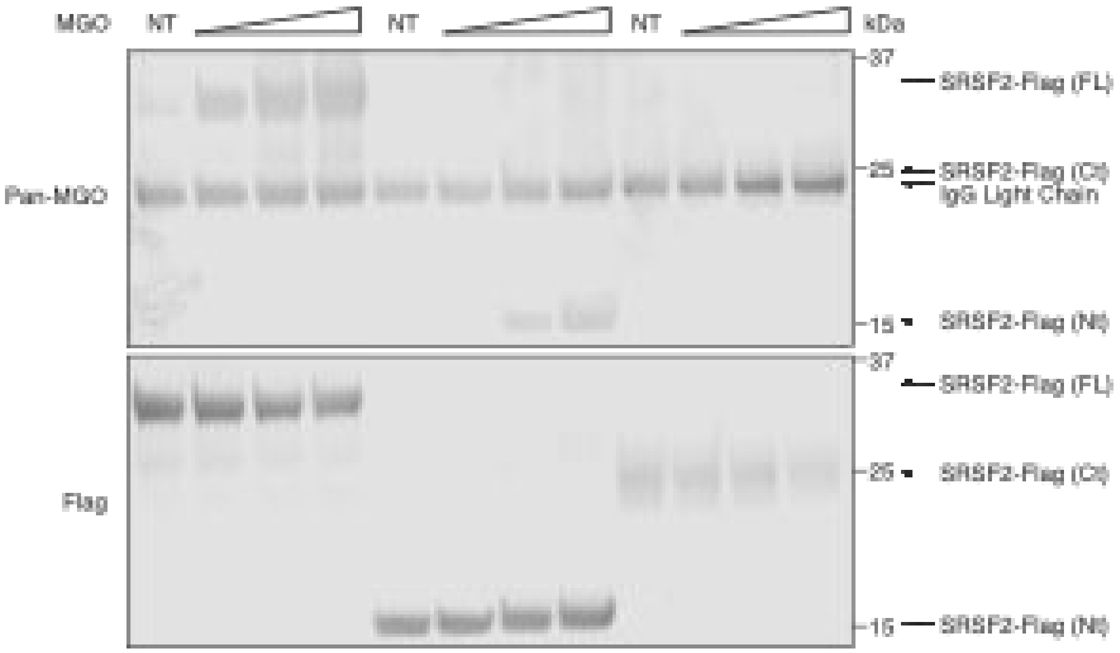

Figure S3. Dose-dependent glycation of SRSF2 full length (FL), N-terminal domain (Nt), and C-terminal domain (Ct) in live cells. HEK293T cells were transfected with FL, Nt, or Ct Flag-tagged SRSF2 and treated with PBS (NT) or increasing concentrations of MGO (0.5-1.5 mM) for 4 h, followed by lysis, Flag-IP, and immunoblotting analysis with anti-pan-MGO and anti-Flag antibodies.

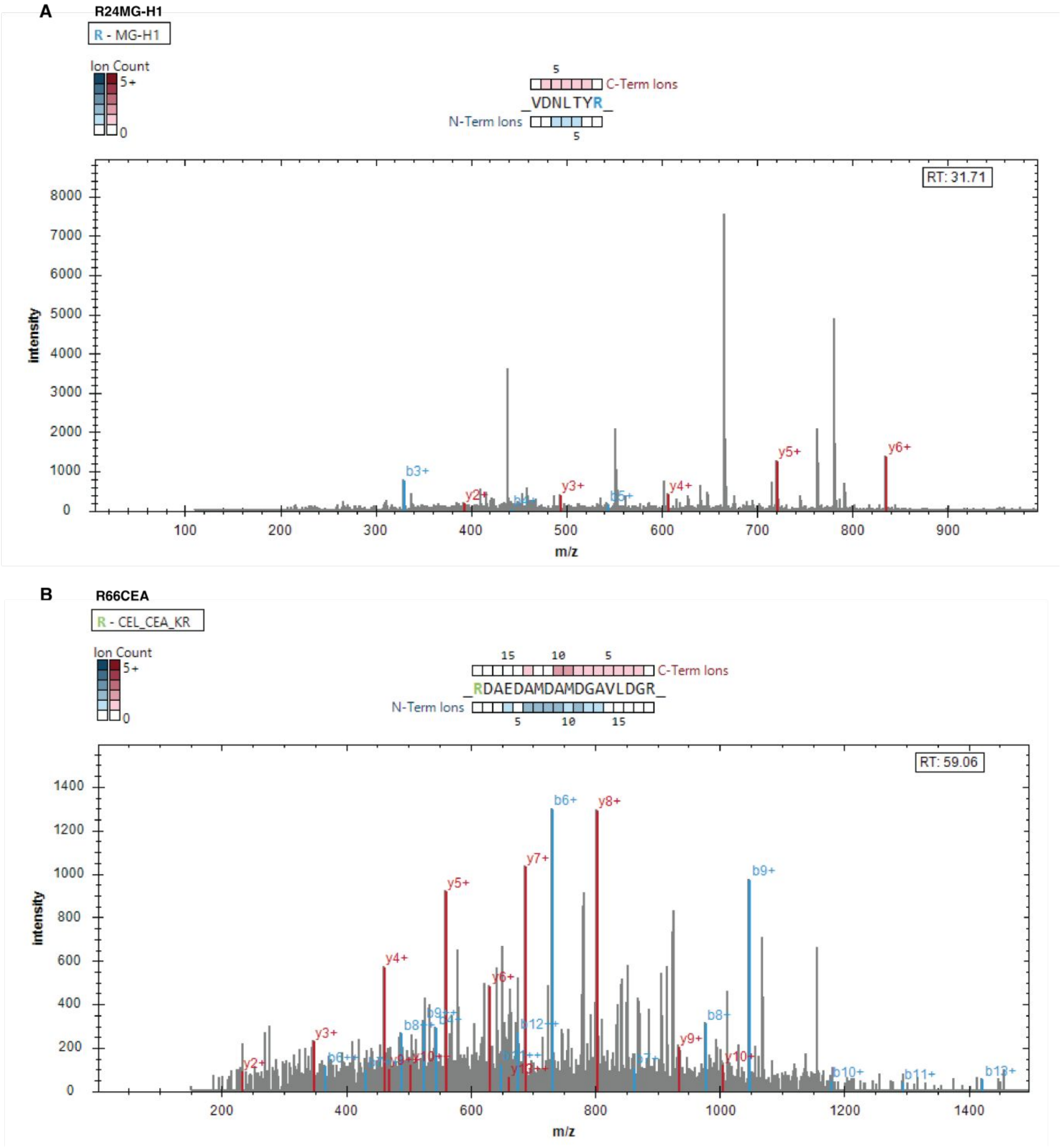

Figure S4. Mapping SRSF2 MGO glycation. MS/MS spectra. (A) DIA-MS spectra of R24MG-H1 glycated peptide. (B) DIA-MS spectra of R66CEA glycated peptide.

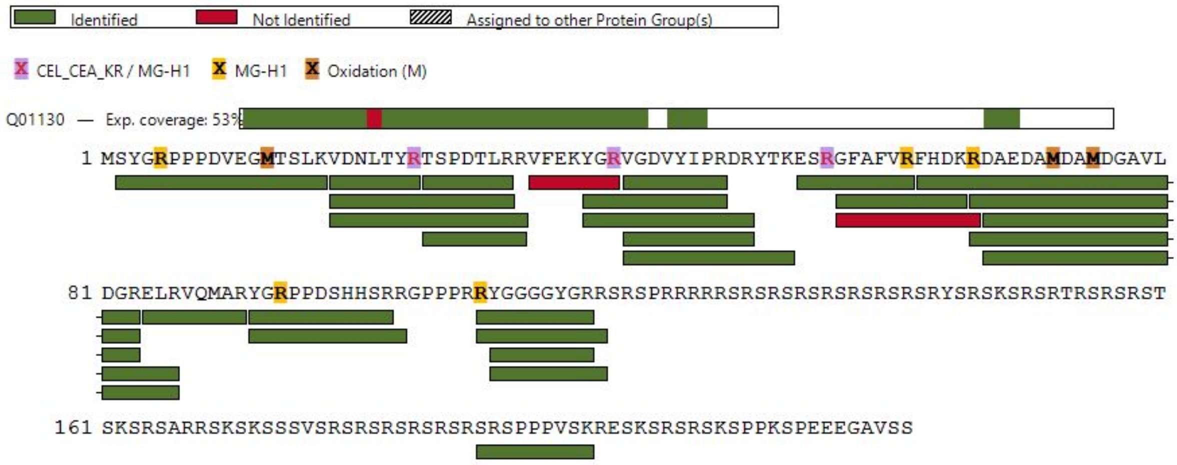

Figure S5. Mass spectrometry coverage of SRSF2 cleaved with trypsin. Green-colored bars correspond to peptide sequences that were detected. CEA ([M+72.02]) and MG-H ([M+54.01]) adducts detected were highlighted in purple and yellow, respectively.

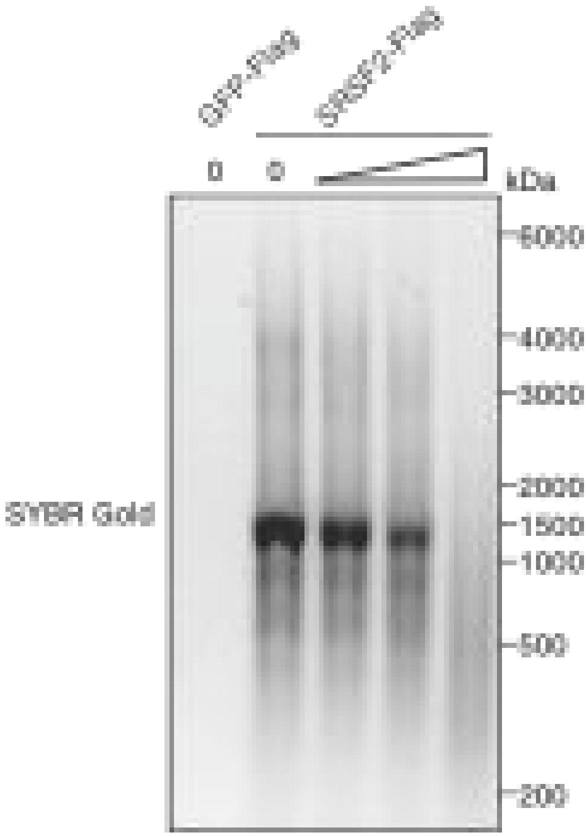

Figure S6. MGO glycation on SRSF2 attenuates its binding to its native RNA substrates. Full-length Flag-tagged glycated SRSF2 was isolated from SRSF2-overexpressing HEK293T cells treated with increasing concentrations of MGO (0-1.5 mM) for 4 h. Isolated SRSF2 was incubated with total RNA from untreated HEK293T cells for RNA immunoprecipitation. GEP-Flag was used as a negative control. The bound RNA was subjected to agarose gel analysis with SYBR gold staining.

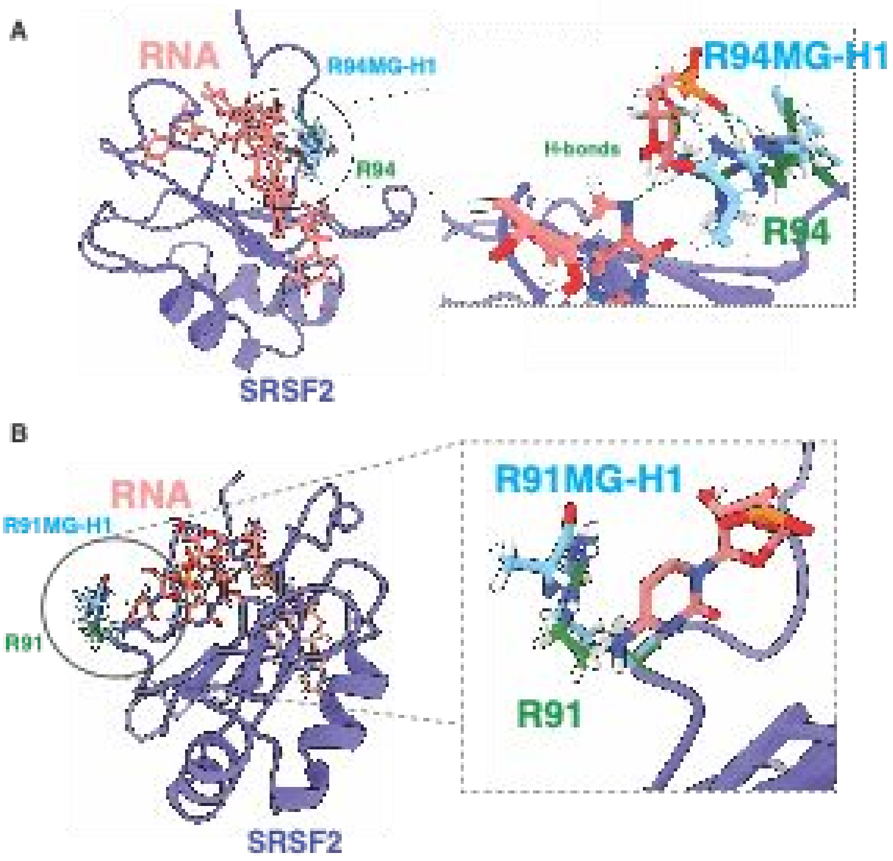

Figure S7. Computational modeling of the impact of site-specific glycation on SRSF2-RNA interaction. (A) R94MG-H1 modelling indicating a sterically clash with the RNA and disruption of hydrogen bonding. (B) R91MG-H1 modelling indicating a sterically clash with the RNA and disruption of hydrogen bonding.

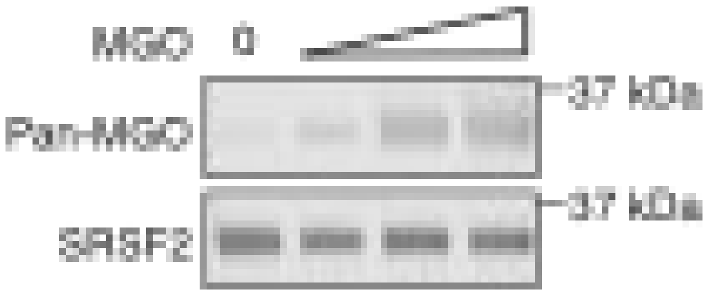

Figure S8. Validation of dose-dependent glycation on Flag-IPed SRSF2 used in RIP-qPCR. Full-length Flag-tagged SRSF2 was isolated from SRSF2-overexpressing HEK293T treated with increasing concentrations of MGO (0-1.5 mM) for 4 h, followed by lysis, Flag-IP, and RIP. 10% of the beads after Flag-IP were subjected to immunoblotting analysis with anti-pan-MGO and anti-SRSF2 antibodies.

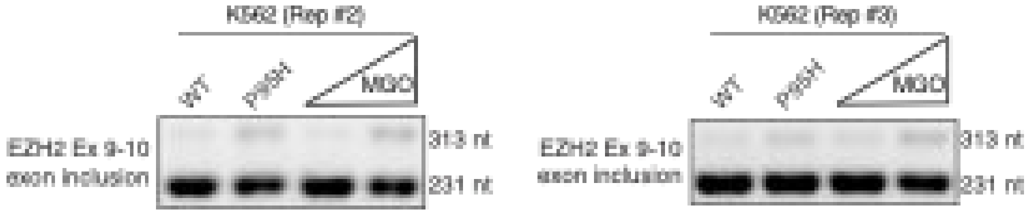

Figure S9. MGO glycation on SRSF2 promotes EZH2 mis-splicing event in K562 cells. Two more independent repeats of the RT-PCR of an EZH2 exon inclusion event in WT SRSF2 K562 cells either treated or untreated with MGO (0.25/0.5 mM) and untreated P95H SRSF2 K562 cells.

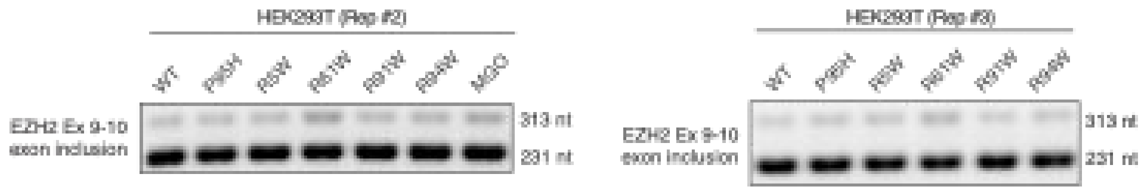

Figure S10. Glycation mimic R-to-W mutant SRSF2 promotes EZH2 mis-splicing event in HEK293T cells. RT-PCR of an EZH2 exon inclusion event in WT SRSF2-overexpressing HEK293T cells either treated or untreated with MGO (0.5 mM) and various untreated R-to-W mutant SRSF2-overexpressing HEK293T cells.

B. General Materials and Methods

General methods (equipment, reagents, chemicals)
UV spectrometry was performed on NanoDrop 2000c (Thermo Scientific). PCR amplifications were performed on a Bio-Rad T100 Thermal Cycler. HPLC Electrospray ionization MS (HPLC-ESI-MS) analysis was performed on an Agilent 6120 Quadrupole LC/MS spectrometer. UPLC–MS was carried out on a Waters Acuity SQD LC-MS in electrospray ionization (ESI) mode. NMR spectra were recorded on a Bruker Avance 400 MHz NMR spectrometer at 24°C in the designated solvents. Biochemicals, chemical reagents, and media were purchased from Fisher Scientific or Sigma-Aldrich Corporation unless otherwise stated. Primer synthesis and plasmid sequencing were performed by Integrated DNA Technologies and Plasmidsaurus, respectively.

Cell culture
All cell lines used in this study were purchased from ATCC unless otherwise stated. HEK293T cell line was a kind gift from the Galligan Lab.[1] SRSF2 WT and P95H K562 cell lines was a kind gift from the Abdel-Wahab Lab.[2] HEK293T cells were cultured in DMEM (4.5 g/L glucose) supplemented with 10% FBS, 2 mM L-glutamine and 500 U mL^{-1} penicillin and streptomycin. K562 cells were cultured in IMDM supplemented with 10% FBS and 500 U mL^{-1} penicillin and streptomycin. All cells were incubated at 37°C with 5% CO_2. All cell lines were monitored for appropriate morphology and tested negative for mycoplasma contamination.

Immunoblotting
Proteins were separated on a 12% Bis-Tris gel with MOPS running buffer (Bio-Rad, 1610788) and transferred to a nitrocellulose membrane using a Criterion blotter system (Bio-Rad). Membranes were blocked with 5% BSA in PBS for 1 h at r.t., then washed (3 x 5 min PBST), and proteins of interest were then probed using the appropriate antibodies for 1 h at r.t. or overnight at 4°C with rocking. Membranes were then washed (3 x 5 min PBST), incubated with secondary antibody (Goat anti-Rabbit IgG, IRDye 800CW (Li-Cor, 926-32213; 1:15000 dilution) and Goat anti-Mouse IgG, IRDye 680CW (Li-Cor, 926-68072; 1:15000 dilution), washed (2 x 5 min PBST, 1 x 5 min PBS), then visualized on a Li-Cor Odyssey CLx fluorescent imaging system.

MGO, AlkMG, and CBR-470-1 treatment
Fresh stock of MGO was prepared by diluting MGO (~40% in H_2O, Sigma, M0252) in PBS before treatment. AlkMG was synthesized and freshly deprotected as described previously.[3,4] Fresh stock of AlkMG was prepared in PBS before treatment. CBR-470-1 (MedChemExpress, HY-134205A) was dissolved in DMSO as a 10 mM stock and diluted in DMSO before treatment.

AlkMG labelling and visualization of AlkMG target proteins
HEK293T cells were grown to 80% confluency in 10-cm plates before being treated with either PBS or 0.1-1.5 mM of AlkMG for 5 h at 37°C. After trypsin treatment, the cells were collected, spun down at 600g for 5 min at 4°C, and washed twice with cold phosphate buffered saline (PBS). The cell pellets were resuspended in RIPA buffer (50 mM HEPES, pH7.4, 150 mM NaCl, 1% sodium deoxycholate, 1% NP-40, 0.5% SDS, 2.5 mM $MgCl_2$, 10 mM sodium glycerophosphate, 10 mM sodium biphosphate) containing protease inhibitor cocktail (Sigma Aldrich, 11836153001) and 25 U/mL benzonase (Millipore, 71205-3). Cell lysates were incubated on ice for 15 min, sonicated on ice twice, and centrifuged at 13000g for 10 min at

4°C. The concentration of the supernatant was measured by BCA assay, and equal amount of lysate was subjected to $CHCl_3$/MeOH precipitation to remove any remaining AlkMG in the cell lysate. The white protein dish was then resuspended in 2% SDS (50 mM HEPES, pH 7.4, 150 mM NaCl, 2.5 mM TCEP) and sonicated once. The resuspended lysate was clicked with biotin alkyne (200 μM Biotin-PEG3-Azide, 500 μM $CuSO_4$, 1 mM THPTA, 5 mM NaAsc, and 5% DMSO) for 3 h at r.t. with rocking. This was followed by $CHCl_3$/MeOH precipitation again to remove excess Biotin-PEG3-Azide, and the lysate was resuspended in 200 μL 2% SDS (50 mM HEPES, pH 7.4, 150 mM NaCl, 2.5 mM TCEP), sonicated, and diluted with RIPA (final SDS < 0.5%). The resulting solution was boiled in 1x SDS loading buffer before loading onto a gel for immunoblotting analysis with IRDye 800CW Streptavidin (Li-Cor, 926-32230; 1:2000 dilution).

AlkMG chemoproteomics

HEK293T cells were grown to 80% confluency in 10-cm plates before being treated with either PBS or 0.1-1.5 mM of AlkMG for 5 h at 37°C. After trypsin treatment, the cells were collected, spun down at 600g for 5 min at 4°C, and washed twice with cold phosphate buffered saline (PBS). The cell pellets were resuspended in RIPA buffer (50 mM HEPES, pH7.4, 150 mM NaCl, 1% sodium deoxycholate, 1% NP-40, 0.5% SDS, 2.5 mM $MgCl_2$, 10 mM sodium glycerophosphate, 10 mM sodium biphosphate) containing protease inhibitor cocktail (Sigma Aldrich, 11836153001) and 25 U/mL benzonase (Millipore, 71205-3). Cell lysates were incubated on ice for 15 min, sonicated on ice twice, and centrifuged at 13000g for 10 min at 4°C. The concentration of the supernatant was measured by BCA assay, and equal amount of lysate was subjected to $CHCl_3$/MeOH precipitation to remove any remaining AlkMG in the cell lysate. The white protein dish was then resuspended in 2% SDS (50 mM HEPES, pH 7.4, 150 mM NaCl, 2.5 mM TCEP) and sonicated once. The resuspended lysate was clicked with biotin alkyne (200 μM Biotin-PEG3-Azide, 500 μM $CuSO_4$, 1 mM THPTA, 5 mM NaAsc, and 5% DMSO) for 3 h at r.t. with rocking. This was followed by $CHCl_3$/MeOH precipitation again to remove excess Biotin-PEG3-Azide, and the lysate was resuspended in 200 μL 2% SDS (50 mM HEPES, pH 7.4, 150 mM NaCl, 2.5 mM TCEP), sonicated, and diluted with RIPA (final SDS < 0.5%). Meanwhile, 20 μL of high-capacity streptavidin agarose beads (Thermo Scientific, 20359) multiplied by the number of samples were washed with 1 mL RIPA and distributed to Eppendorf tubes. The lysates were added to the beads and incubated overnight at RT on a rotator. The beads were washed with RIPA ×2, 1 M KCl, 0.1 M Na_2CO_3, 2 M urea in HEPES buffer ×2, RIPA ×2, and water x3. After the final wash with water, the samples were quantified using a BCA assay. For this experiment, 200 μg of proteins were digested by trypsin. After protein digestion, approximately 100 μg of peptides were labeled using a TMT chemical labeling approach with a TMT 11 plex kit. The TMT incorporation efficiency was evaluated via ratio-check. Peptides were fractionated into 8 fractions using high-pH fractionation and combined into 4 fractions. The samples were run on an Eclipse mass spectrometer using a TMT pro SPS MS3 method, coupled to a Neo Vanquish liquid chromatography system, using a 4-hour gradient. For quality control, standards were run before and after the project to check instrument conditions. The mass spectrometry runs, along with the number of peptides and proteins identified and quantified, were consistent with expectations for these types of experiments. The data analyses were performed with Proteome Discoverer version 2.4.0.305 (Thermo Scientific). Raw files from all fractions were merged and searched with the SEQUEST HT search engine with a Homo Sapien SwissProt protein database downloaded on 2024/02/20 (42,428 entries). Methionine oxidation was set as variable modification, while Cys carbamidomethylation, TMT6plex (K) and TMT6plex (N-term) were specified as fixed modifications. The precursor and fragment mass tolerances were 10 ppm and 0.6 Da respectively. A maximum of two trypsin missed cleavages were permitted. Searches

used a reversed sequence decoy strategy to control peptide false discovery rate (FDR) and 1% FDR was set as threshold for identification. Proteins were considered enriched if they have a fold-change(AlkMG/MGO)≥2.0 and p-value≤0.05. Gene set enrichment analysis (GSEA, http://software.broadinstitute.org/gsea) was run against MSigDB v6 using the pre-ranked option and log2 fold change for pairwise comparisons.

AlkMG target validation

The same treatment, click labeling, and enrichment protocol from AlkMG chemoproteomics was used here. After the final wash, the streptavidin beads were boiled in 1x SDS loading buffer before loading onto a gel for immunoblotting analysis with anti-SRSF2 (abcam, ab204916; 1:1000 dilution), anti-SRSF1 (Invitrogen, 32-4500; 2 μg/mL), anti-HMGA1 (Cell Signaling Technology, 7777; 1:1000 dilution), and anti-MCM2 (Cell Signaling Technology, 12079S; 1:1000 dilution).

Purification of recombinant SRSF2 RRM (AA 1-101)

The pET26b(+)-SRSF2(AA 1-101)-6xHis expression vector was a kind gift from the Manley Lab.[5] Protein expression was induced in Rosetta(DE3) cells at 20°C overnight by 1 mM isopropyl β-D-1-thiogalactopyranoside. Proteins were purified with nickel affinity chromatography using Ni-NTA Resin (Thermo Scientific, 88223) as previously described. Proteins were dialyzed into a buffer containing 5mM Na_2HPO_4 and 15mM NaH_2PO_4, pH=5.5 once at r.t. for 2 h and then overnight at 4°C, and then were concentrated using Amicon Ultra centrifugal filters (3 kDa MWCO, Merck Millipore, UFC900324). Protein concentrations were determined by measuring their optical density at 280 nm.

***In vitro* glycation**

Recombinant SRSF2 glycation assays were prepared on ice and contained 20 μM of the recombinant SRSF2 RRM (AA 1-101) in PBS buffer (pH 7.4) and 0, 0.1, 0.5, 1.0, 2.5, 5.0, 10.0, or 20.0 mM of MGO. Reactions were incubated at 37°C for 2 h and quenched with 10 mM Tris. Samples were boiled in 1x SDS loading buffer before loading onto a gel for immunoblotting analysis with pan-MGO (Cell Biolabs, STA-011; 1:2000 dilution) and anti-SRSF2 (abcam, ab204916; 1:1000 dilution).

***In cellulo* glycation**

The pCMV-SRSF2-Flag plasmid was a kind gift from the Fuks Lab.[6] The Flag-tagged full-length SRSF2 was overexpressed in HEK293T cells using Lipofectamine 2000 Transfection Reagent (Thermo Fisher Scientific, 11668019) according to the manufacturer's protocol. One day after the transfection, HEK293T cells were treated with 0, 0.5, 1.0 or 1.5 mM MGO for 4 h. The harvested cell pellet was reconstituted in the lysis buffer (50 mM Tris, pH 7.4, 150 mM NaCl, 1 mM EDTA, 1% Triton X-100) containing protease inhibitor cocktail (Sigma Aldrich, 11836153001), incubated on ice for 5 min, sonicated, cleared at 13000g for 10 min at 4°C. The supernatant containing the whole cell lysate was transferred to a new tube and the concentration was measured using a BCA assay. Equal amount of lysate was incubated with 50 μL pre-washed anti-FLAG M2 Affinity Gel (Sigma, A2220) at 4°C for 2 h, followed by 3 washes with 0.5 mL lysis buffer (with 500 mM NaCl). The beads were then boiled in 1x sample loading buffer before loading onto a gel for immunoblotting analysis with pan-MGO (Cell Biolabs, STA-011; 1:2000 dilution) and anti-SRSF2 (abcam, ab204916; 1:1000 dilution).

Data-independent acquisition (DIA) IP-MS identification of SRSF2 glycation sites

The Flag-tagged full-length SRSF2 was overexpressed in HEK293T cells using Lipofectamine 2000 Transfection Reagent (Thermo Fisher Scientific, 11668019) according to the

manufacturer's protocol. One day after the transfection, HEK293T cells were treated with 0, 0.5, 1.0 or 1.5 mM MGO for 4 h. The harvested cell pellet was reconstituted in the lysis buffer (50 mM Tris, pH 7.4, 150 mM NaCl, 1 mM EDTA, 1% Triton X-100) containing protease inhibitor cocktail (Sigma Aldrich, 11836153001), incubated on ice for 5 min, sonicated, cleared at 13000g for 10 min at 4°C. The supernatant containing the whole cell lysate was transferred to a new tube and the concentration was measured using a BCA assay. Equal amount of lysate was incubated with 50 µL pre-washed anti-FLAG M2 Affinity Gel (Sigma, A2220) at 4°C for 2 h, followed by 3 washes with 0.5 mL lysis buffer (with 500 mM NaCl) and 2 washes with PBS. The IPed samples on beads were reduced with DTT, alkylated with IAA, and digested with trypsin overnight at 37℃. Peptides were then desalted using C18 StageTips, dried by vacuum centrifugation, and reconstituted in 12 µL 0.1% formic acid. Samples were then transferred to LC vials and set up for LC/MS-MS. The samples were run on an Eclipse mass spectrometer using a TMT pro SPS MS3 method, coupled to a Neo Vanquish liquid chromatography system, using a 4-hour gradient. Raw data files were processed using Spectronaut version 19.1 (Biognosys) and searched with the PULSAR search engine with a Homo Sapiens SwissProt protein database downloaded on 2024/05/28 (226,232 entries). Cysteine carbamidomethylation was set as fixed modification, while methionine oxidation, protein N-terminus acetylation, and deamidation (NQ), MG-H1(R), CEL(K), and CEA (R) were set as variable modifications.[1,7] A maximum of two trypsin missed cleavages were permitted. Searches used a reversed sequence decoy strategy to control peptide false discovery rate (FDR), with a threshold of 1% FDR for identification.

Co-immunoprecipitation

The GFP sequence was cloned into the pCMV plasmid by Gibson assembly. The Flag-tagged full-length SRSF2 or GFP were overexpressed in HEK293T cells using Lipofectamine 2000 Transfection Reagent (Thermo Fisher Scientific, 11668019) according to the manufacturer's protocol. One day after the transfection, HEK293T cells were treated with 0, 0.5, 1.0 or 1.5 mM MGO for 4 h. The harvested cell pellet was reconstituted in the lysis buffer (50 mM Tris, pH 7.4, 150 mM NaCl, 1 mM EDTA, 1% Triton X-100) containing protease inhibitor cocktail (Sigma Aldrich, 11836153001), incubated on ice for 5 min, sonicated, cleared at 13000g for 10 min at 4°C. The supernatant containing the whole cell lysate was transferred to a new tube and the concentration was measured using a BCA assay. Equal amount of lysate was incubated with 50 µL pre-washed anti-FLAG M2 Affinity Gel (Sigma, A2220) at 4°C for 2 h, followed by 3 washes with 0.5 mL lysis buffer (with 500 mM NaCl). The beads with protein bound were transferred into 0.5 mg of non-treated HEK293T whole cell lysate and incubated at 4℃ for 2 h. The beads were then washed 0.5 mL lysis buffer three times and boiled in 1x sample loading buffer before loading onto a gel for immunoblotting analysis with anti-snRNP70 (Santa Cruz Biotechnology, sc-390899; 1:500 dilution), anti-U2AF1 (abcam, ab86305; 1:2000 dilution), pan-MGO (Cell Biolabs, STA-011; 1:2000 dilution) and anti-SRSF2 (abcam, ab204916; 1:1000 dilution).

Biolayer interferometry

Biolayer interferometry (BLI) binding data were collected on an Octet R8 (Sartorius) and processed using the instrument's integrated software. Recombinant SRSF2 glycation assays were prepared on ice and contained 36 µM of the recombinant SRSF2 RRM (AA 1-101) in PBS buffer (pH 7.4) and either PBS or 2.5 mM of MGO. Reactions were incubated at 37°C for 1 h. The protein solution was then buffer exchanged into the BLI buffer (20 mM NaH_2PO_4, pH 5.5, 0.005% Tween, 1% BSA) with 7K MWCO Zeba Micro Spin Desalting Columns (Thermo Scientific, 89877) and nanodropped to normalized protein concentrations across samples. For SRSF2-RNA binding assays, 10 nM biotinylated CCGG-containing RNA oligo (5'-

UUUCAGCUCCGGUCACGCUC-biotin-3’, purchased from Integrated DNA Technologies) were loaded onto streptavidin-coated biosensors (Sartorius, 18-5019) in BLI buffer for 6 min. Unmodified or glycated SRSF2 were diluted from concentrated stocks into the BLI buffer. After baseline measurement in the binding buffer alone, the binding kinetics were monitored by dipping the biosensors in wells containing the target protein at the indicated concentration (association step) and then dipping the sensors back into baseline/buffer (dissociation). Data were analyzed and processed using Octet Analysis Studio v.13.0.3.52.

***In vitro* RNA pull-down**

The Flag-tagged full-length SRSF2 or GFP were overexpressed in HEK293T cells using Lipofectamine 2000 Transfection Reagent (Thermo Fisher Scientific, 11668019) according to the manufacturer’s protocol. One day after the transfection, HEK293T cells were treated with 0, 0.5, 1.0 or 1.5 mM MGO for 4 h. The harvested cell pellet was reconstituted in the lysis buffer (50 mM HEPES, pH 7.6, 150 mM NaCl, 1 mM EDTA, 1% Triton X-100, 1.5 mM $MgCl_2$) containing protease inhibitor cocktail (Sigma Aldrich, 11836153001) and 25 U/mL benzonase (Millipore, 71205-3), incubated on ice for 5 min, sonicated, cleared at 13000g for 10 min at 4°C. The supernatant containing the whole cell lysate was transferred to a new tube and the concentration was measured using a BCA assay. Equal amount of lysate was incubated with 50 µL pre-washed anti-FLAG M2 Affinity Gel (Sigma, A2220) at 4°C for 2 h, followed by 3 washes with 0.5 mL lysis buffer (with 500 mM NaCl). The beads with protein bound were then equilibrated in binding buffer (50 mM Tris, pH 7.4, 250 mM NaCl, 0.4 mM EDTA, 0.1% NP-40) with RNase inhibitor (Invitrogen, AM2696). 2.5 µg Cy5-labelled CCUG-containing RNA oligo (5’-UGUAGUAGUUCAGCCUGGU-Cy5-3’, purchased from Integrated DNA Technologies) was added to each IP reaction and incubated for 2 h at 4°C. The beads were then washed three times with ice cold binding buffer, and 10% of the beads were transferred to a new tube for immunoblotting analysis with ponceau staining for equal loading. The remaining 90% of the beads were incubated with 4 mg/mL Proteinase K for 1 h at55°C. The resulting supernatant was transferred to a new tube and purified via the Monarch RNA Cleanup Kit (T2030). The eluted RNA was subjected to a 3% agarose gel and quantified by the Cy5 fluorescence signal.

RNA immunoprecipitation (RIP)-qPCR

The RIP experimental procedure was adapted from the previously reported method.[6] The Flag-tagged full-length SRSF2 or GFP were overexpressed in HEK293T cells using Lipofectamine 2000 Transfection Reagent (Thermo Fisher Scientific, 11668019) according to the manufacturer’s protocol. One day after the transfection, HEK293T cells were treated with 0, 0.5, 1.0 or 1.5 mM MGO for 4 h. The harvested cell pellet was reconstituted in the lysis buffer (50 mM HEPES, pH 7.6, 150 mM NaCl, 1 mM EDTA, 1% Triton X-100, 1.5 mM $MgCl_2$) containing protease inhibitor cocktail (Sigma Aldrich, 11836153001) and 25 U/mL benzonase (Millipore, 71205-3), incubated on ice for 5 min, sonicated, cleared at 13000g for 10 min at 4°C. The supernatant containing the whole cell lysate was transferred to a new tube and the concentration was measured using a BCA assay. Equal amount of lysate was incubated with 50 µL pre-washed anti-FLAG M2 Affinity Gel (Sigma, A2220) at 4°C for 2 h, followed by 3 washes with 0.5 mL lysis buffer (with 500 mM NaCl). The beads with protein bound were then equilibrated in RIP buffer (50 mM HEPES, pH 7.6, 250 mM NaCl, 5 mM EDTA, 0.05% NP-40) with protease inhibitor cocktail (Sigma Aldrich, 11836153001) and 40 U/mL RNase inhibitor (Invitrogen, AM2696). Total RNA was isolated from untreated HEK293T cells with the RNeasy Plus Kit (Qiagen, 74134). 30 µg of total RNA was added to each RIP reaction and incubated for 45 min at r.t.. The beads were then washed five times with ice cold RIP buffer, and 10% of the beads were transferred to a new tube for immunoblotting analysis with pan-

MGO (Cell Biolabs, STA-011; 1:2000 dilution) and anti-SRSF2 (abcam, ab204916; 1:1000 dilution). The remaining 90% of the beads were incubated with 4 mg/mL Proteinase K for 1 h at 55°C. The resulting supernatant was transferred to a new tube and purified via the Monarch RNA Cleanup Kit (T2040). The eluted RNA was subjected to a 1% agarose gel analysis with SYBR Gold staining. For RT-qPCR, the input and eluted RNA were treated with RNase-Free DNase (Promega, M6101) to remove the residual DNA. 2 µg of DNA-free RNA was reverse transcribed using the High-Capacity RNA-to-cDNA Kit (Thermo Fisher Sceintific, 4387406). qPCR was performed for each cDNA (~1 ng) sample in triplicate using the iTaq Universal SYBR Green Supermix (Bio-Rad, 1725122). The RT-qPCR data were presented as the %input in mRNA abundance in the RIPed sample relative to the input. The sequences of primers used in qPCR were:

EZH2: 5'-TTCATGCAACACCCAACACT-3' (forward) and 5'-GAGAGCAGCAGCAAACTCCT-3' (reverse)
INTS3: 5'-GGTACGGGAACTGGTGAAGA-3' (forward) and 5'-CTGCTCTTCAGGACCCACTC-3' (reverse)
UBN1: 5'-CGATGCCAGAGCAGATGGC-3' (forward) and 5'-GAACTTCTTCCGAGGTCCCATTATCC-3' (reverse)
YWHAZ: 5'-CCGCTGGTGATGACAAGAAAGGGAT-3' (forward) and 5'-AGGGCCAGACCCAGTCTGATAGGA-3' (reverse)

Structure-based biophysical computational modeling
To enable accurate structure-based modeling of post-translational modifications, we parameterized methylglyoxal-derived hydroimidazolone-1 (MG-H1) as a non-canonical amino acid (NCAA) in Rosetta.[8,9] A dipeptide structure of MG-H1 was generated and exported in molfile (.sdf) format. The N- and C-termini were capped with methyl groups to represent adjacent Cα atoms, ensuring correct backbone connectivity during Rosetta polymerization. The .sdf file contained explicit annotations defining backbone atoms (N, CA, C, O), connection points for polymerization, and stereochemical assignments. In addition to backbone connectivity, two key chemical properties were encoded in the input file: (1) MG-H1's net zero formal charge, reflecting the loss of the positively charged guanidinium group upon glycation, and (2) the presence of a heterocyclic ring in the side chain. These features were included using Rosetta-compatible annotations in the .sdf file to ensure correct electrostatics and side chain geometry during parameterization and subsequent simulations.

Because MG-H1 retains the backbone and much of the sidechain geometry of arginine, we employed arginine as the parent residue for rotamer library inheritance. A Rosetta parameter file (.params) was generated using the molfile_to_params_polymer.py script:

```
python $ROSETTA/main/source/scripts/python/public/molfile_to_params_polymer.py \
  --clobber --polymer --no-pdb --name MGH1 --use-parent-rotamers ARG \
  -i ../input_files/MGH1.sdf
```

To quantify the energetic impact of arginine glycation on SRSF2–RNA interactions, we applied a previously established Rosetta–Vienna ΔΔG protocol for protein–RNA complexes.[10] The RNA-binding protein SRSF2 was modeled in complex with its canonical RNA motif, uCCAGu, which represents one of the most frequently recognized binding sequences for SRSF2.[11] Three-dimensional structures of WT SRSF2 in complex with the uCCAGu RNA motif were generated and minimized prior to energy calculations. For the modified system, each arginine residue positioned at the RNA interface (R5, R61, R91, and R94) was individually substituted

with MG-H1 using the previously generated NCAA parameter file (MGH1.params). This allowed direct energetic comparison between unmodified and glycated states while preserving backbone geometry and local structural context. The structure to perform simulations in this study was PDB: 2LEB.[11]

Binding free energies (ΔG) for both WT and MG-H1–modified SRSF2–RNA complexes were computed using the Rosetta–Vienna RNP ΔΔG protocol, which is specifically optimized for protein nucleic acid interfaces. Briefly, the protein–RNA complexes were relaxed and energy-minimized using the Rosetta rnp_ddg weight set to ensure accurate scoring of both protein and RNA contributions. For each structure (WT or MG-H1 mutant), the total energy of the bound complex and the energies of the unbound partners (protein and RNA after rigid-body separation) were computed. The difference between these energies yielded the binding free energy:

$$\Delta G_{(\frac{WT}{MUT})} = E_{bound} - E_{protein} - E_{RNA}$$

This was repeated 100x times, and top 40 complexes with lowest rosetta energy were selected. This procedure was repeated for the MG-H1–modified complexes, maintaining identical backbone coordinates to isolate the energetic contribution of the chemical modification. Structures were visualized and molecular representations generated using ChimeraX.

Site-directed mutagenesis

The pCMV-SRSF2-Flag and pCMV-SRSF2^{P95H}-Flag plasmids were a kind gift from the Fuks Lab.[6] The R-to-W mutant plasmids were generated by introducing point mutations (either R5W, R61W, R91W, or R94W) into WT SRSF2 plasmid using the Q5 Site-Directed Mutagenesis Kit (NEB, E0552S) according to the manufacturer's instructions. All plasmids were verified by whole plasmid sequencing performed by Plasmidsaurus. The sequences of primers used were:

R5W: 5'-GAGCTACGGCtggCCCCCTCCCG-3' (forward) and 5'-ATGGGCCCGCGGGTACAATTC-3' (reverse)
R61W: 5'-CGCCTTCGTTtggTTTCACGACAAG-3' (forward) and 5'-AAGCCGCGGGACTCC-3' (reverse)
R91W: 5'-GCAAATGGCGtggTACGGCCGCC-3' (forward) and 5'-ACCCGCAGCTCGCGG-3' (reverse)
R94W: 5'-GCGCTACGGCtggCCCCCGGACT-3' (forward) and 5'-GCCATTTGCACCCGCAGCTCGC-3' (reverse)

RT-PCR

Total RNA was extracted from SRSF2 WT cells (treated with PBS or 0.25/0.5 mM MGO overnight), SRSF2 P95H K562 cells, HEK293T cells overexpressing SRSF2 WT (treated with PBS or 0.5 mM MGO overnight), or HEK293T cells overexpressing various SRSF2 mutants with the RNeasy Plus Kit (Qiagen, 74134). 1 µg of RNA was reverse transcribed using the Verso cDNA synthesis Kit (Thermo Scientific, AB1453B). PCR was performed for each cDNA (~70 ng) sample using the GOTaq Master Mix (Promega, M7122). The resulting samples were subjected to a 1.5% agarose gel and visualized with ethidium bromide. The sequences of primers used in PCR were:

EZH2 Ex 9-10 exon inclusion: 5'-GGAAGAACACAGAAACAGCTC-3' (forward) and 5'-CTGCTTCCCTATCACTGTCTG-3' (reverse)

EZH2 immunoblotting

SRSF2 WT K562 cells were cultured in either low-glucose (5 mM) or high-glucose (25 mM) IMDM media for 2 passages. SRSF2 P95H K562 cells were only cultured in low-glucose media. WT cells were treated with either PBS, 20 μM CBR-470-1 or 0.1 mM MGO overnight. The harvested cell pellet was reconstituted in the lysis buffer (50 mM Tris, pH 7.4, 150 mM NaCl, 1 mM EDTA, 1% Triton X-100) containing protease inhibitor cocktail (Sigma Aldrich, 11836153001), incubated on ice for 5 min, sonicated, cleared at 13000g for 10 min at 4°C. The supernatant containing the whole cell lysate was transferred to a new tube and the concentration was measured using a BCA assay. Equal amount of lysate was boiled in 1x sample loading buffer before loading onto a gel for immunoblotting analysis with anti-EZH2 (Cell Signaling Technology, 5246S; 1:1000 dilution) and anti-beta Actin (abcam, ab8226; 1:1000 dilution). EZH2 level in each condition was quantified by normalizing the EZH2 band intensity to the loading control beta Actin.

C. Compound Synthesis and Characterization

Scheme S1. Synthesis of 1,1-diethylacetal-protected AlkMG (4) probe

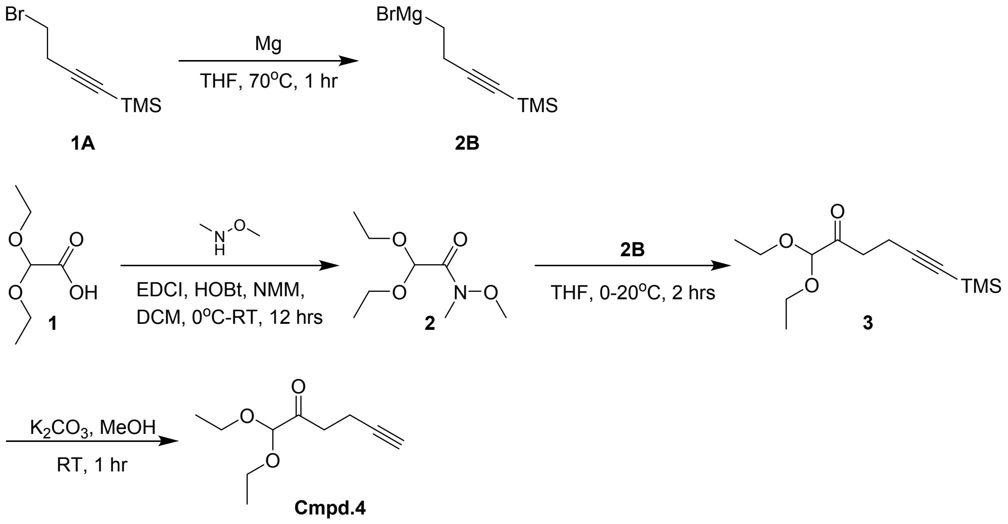

Bromo(4-trimethylsilylbut-3-ynyl)magnesium (2B)

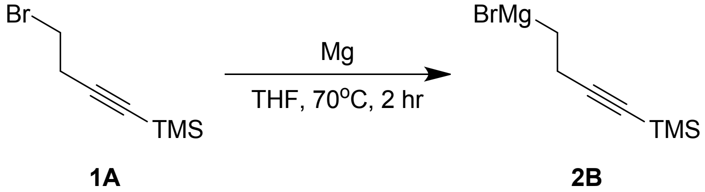

To a solution of Mg (4.62 g, 190.09 mmol, 3 eq) in THF (40 mL) was added 4-bromobut-1-ynyl(trimethyl)silane (1.3 g, 6.336 mmol, 0.1 eq) in THF (1 mL) under N_2 atmosphere. The Grignard reaction was initiated by heating the flash slightly with a heat gum until reflux. The mixture was stirred at 70°C for 1 h under N_2 atmosphere. Then a solution of 4-bromobut-1-ynyl(trimethyl)silane (13 g, 63.36 mmol, 1 eq) in THF (10 mL) was added to the mixture and stirred at 70°C for 1 h until most Mg had disappeared. The solution of bromo(4-trimethylsilylbut-3-ynyl)magnesium (14.5 g, 1.2M in THF) was used into the next step.

2,2-Diethoxy-N-methoxy-N-methyl-acetamide (2)

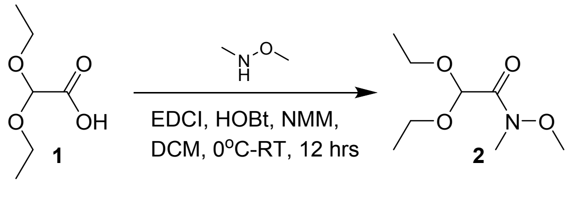

To a solution of 2,2-diethoxyacetic acid (5 g, 33.75 mmol, 1 eq) and N-methoxymethanamine (4.12 g, 67.50 mmol, 2 eq) in DCM (100 mL) was added EDCI (9.70 g, 50.62 mmol, 1.5 eq), NMM (17.07 g, 168.74 mmol, 18.55 mL, 5 eq) and HOBt (6.84 g, 50.62 mmol, 1.5 eq) at 0°C. The mixture was stirred at 25°C for 12 h. The reaction mixture was quenched by 1M HCl (30 mL) at 0°C, and extracted with EtOAc 60 mL (3 x 20 mL). The combined organic layers were dried over Na_2SO_4, filtered and concentrated under reduced pressure to give a residue. The

residue was purified by column chromatography (silica gel, 100-200 mesh, 0-100% ethyl acetate in petroleum ether) affording 2,2-diethoxy-N-methoxy-N-methyl-acetamide (5 g, 26.15 mmol, 77.48% yield) as a yellow oil. ^1H NMR (400 MHz, $CDCl_3$): δ 5.31 - 5.22 (m, 1H), 3.71 - 3.54 (m, 7H), 3.14 (br s, 3H), 1.19 (t, J = 7.1Hz, 6H). ESI-MS: calcd for $C_8H_{18}NO_4$ $[M+H]^+$ 192.12; found 192.1.

1,1-Diethoxy-6-trimethylsilyl-hex-5-yn-2-one (3)

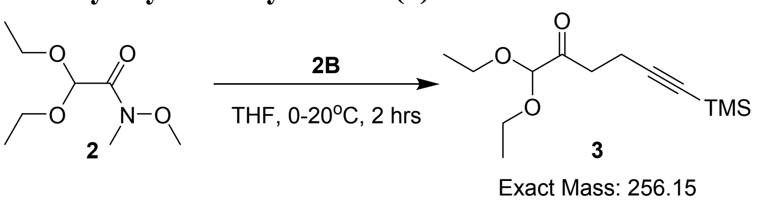

To a solution of 2,2-diethoxy-N-methoxy-N-methyl-acetamide (1.4 g, 7.32 mmol, 1 eq) in THF (10 mL) was added bromo(4-trimethylsilylbut-3-ynyl)magnesium (1.2 M, 42.71 mL, 7 eq) (in THF) at 0°C. The mixture was stirred at 20°C for 1 h under N_2 atmosphere. The reaction mixture was quenched by saturated NH_4Cl (10 mL) at 0°C, then the mixture was concentrated under reduced pressure to remove THF. The mixture was extracted with MTBE (30 mL) (3 x 10 mL). The combined organic layers were dried over Na_2SO_4, filtered and concentrated under reduced pressure to give a residue. The residue was purified by column chromatography (silica gel, 100-200 mesh, 0-100% ethyl acetate in petroleum ether) affording 1,1-diethoxy-6-trimethylsilyl-hex-5-yn-2-one (2 g, 7.80 mmol, 35.51% yield) as a yellow oil. ^1H NMR (400 MHz, $CDCl_3$): δ 4.59 (s, 1H), 3.76 - 3.67 (m, 2H), 3.59 (br d, J = 7.1 Hz, 2H), 2.87 (t, J =7.5 Hz, 2H), 2.50 (t, J = 7.5 Hz, 2H), 1.26 (t, J = 7.1 Hz, 6H), 0.18 - 0.12 (m, 9H). ESI-MS: calcd for $C_{13}H_{25}O_3Si$ $[M+H]^+$ 257.15; found 257.6.

1,1-diethoxyhex-5-yn-2-one (4)

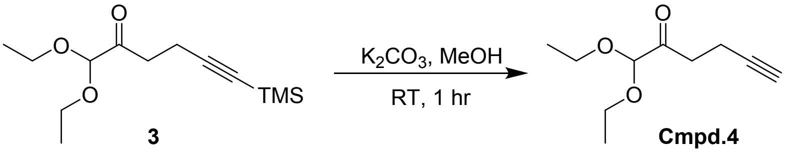

To a solution of 1,1-diethoxy-6-trimethylsilyl-hex-5-yn-2-one (1.5 g, 5.85 mmol, 1 eq) in MeOH (10 mL) was added K_2CO_3 (1.21 g, 8.77 mmol, 1.5 eq). The mixture was stirred at 30°C for 1 h. The reaction mixture was mixed with H_2O (15 mL), and extracted with DCM (60 mL) (3 x 20 mL). The combined organic layers were dried over Na_2SO_4, filtered and concentrated under reduced pressure to give a residue. The residue was purified by column chromatography (silica gel, 100-200 mesh, 0-100% ethyl acetate in petroleum ether) affording 1,1-diethoxyhex-5-yn-2-one (750 mg, 4.07 mmol, 69.59% yield) as a yellow oil. ^1H NMR (400 MHz, $CDCl_3$): δ 4.48 (s, 1H), 3.65 - 3.55 (m, 2H), 3.52 - 3.41 (m, 2H), 2.76 (t, J = 7.3 Hz, 2H), 2.35 (dt, J = 2.6, 7.3 Hz, 2H), 1.84 (t, J = 2.6 Hz, 1H), 1.15 (t, J = 7.1 Hz, 6H). ESI-MS: calcd for $C_{10}H_{17}O_3$ $[M+H]^+$ 185.12; found 185.2.

Deprotection of 4 for AlkMG

Removal of the acetal was carried out immediately before treatment. 50 mg of 1,1-diethoxyhex-5-yn-2-one was suspended in 1 mL H_2O in a glass vial. 100 mg Dowex 50WX8100 cation exchange resin was added to the solution, and the mixture was stirred at 100°C for 30 min in an oil bath. TLC (50% EtOAc in hexane) indicated full conversion of the starting material. The solution was then filtered through a cotton plug to remove the resin. The filtrate was analyzed

by a previously reported quantification method[12] with 3,4-diaminobenzophenone (DABP). Briefly, 100 µL of a 20 mM stock of DABP dissolved in DMF was added to 100 µL of a 2 mM AlkMG solution in 20 mM PBS and incubated at 37°C for 30 min. The AlkMG-DABP addition product was confirmed by UPLC-MS. ESI-MS: calcd for $C_{19}H_{15}N_2O$ $[M+H]^+$ 287.12; found 287.17.

^{1}H-NMR spectrum of 4

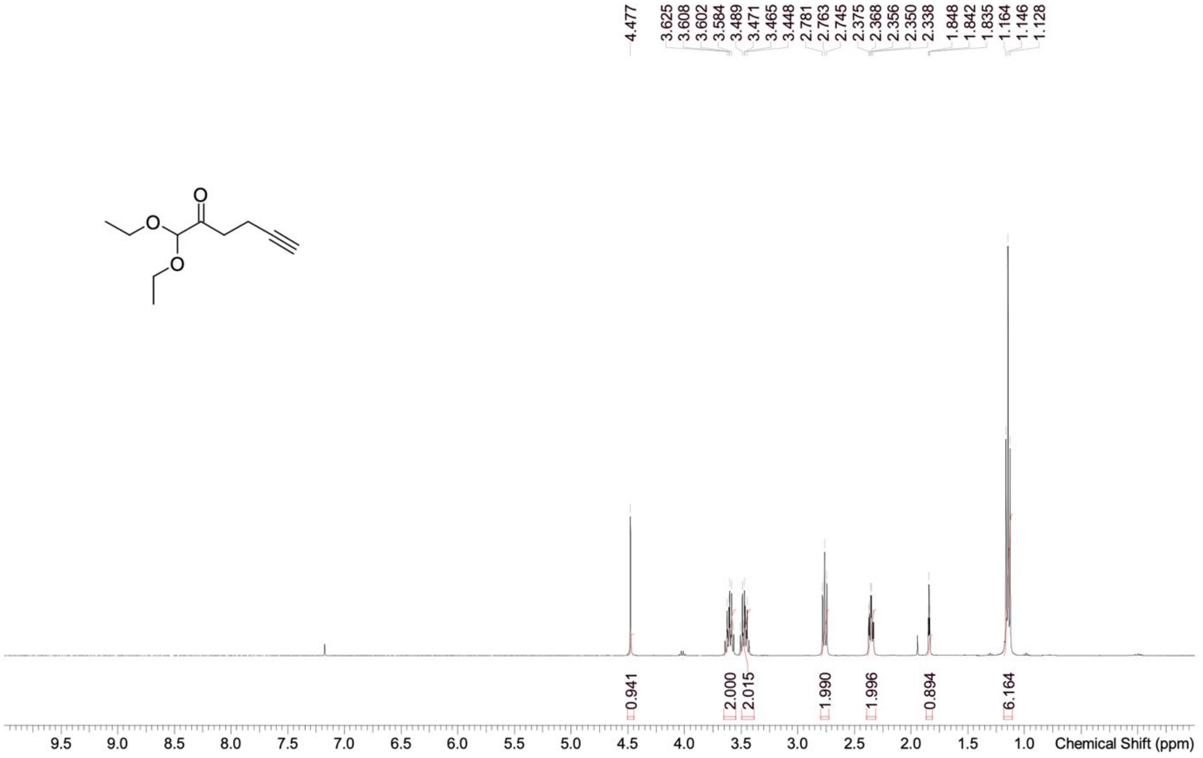

D. References

(1) Galligan, J. J.; Wepy, J. A.; Streeter, M. D.; Kingsley, P. J.; Mitchener, M. M.; Wauchope, O. R.; Beavers, W. N.; Rose, K. L.; Wang, T.; Spiegel, D. A.; Marnett, L. J. Methylglyoxal-Derived Posttranslational Arginine Modifications Are Abundant Histone Marks. *Proc. Natl. Acad. Sci.* **2018**, *115* (37), 9228–9233.

(2) Kim, E.; Ilagan, J. O.; Liang, Y.; Daubner, G. M.; Lee, S. C.-W.; Ramakrishnan, A.; Li, Y.; Chung, Y. R.; Micol, J.-B.; Murphy, M. E.; Cho, H.; Kim, M.-K.; Zebari, A. S.; Aumann, S.; Park, C. Y.; Buonamici, S.; Smith, P. G.; Deeg, H. J.; Lobry, C.; Aifantis, I.; Modis, Y.; Allain, F. H.-T.; Halene, S.; Bradley, R. K.; Abdel-Wahab, O. SRSF2 Mutations Contribute to Myelodysplasia by Mutant-Specific Effects on Exon Recognition. *Cancer Cell* **2015**, *27* (5), 617–630.

(3) Zheng, Q.; Maksimovic, I.; Upad, A.; Guber, D.; David, Y. Synthesis of an Alkynyl Methylglyoxal Probe to Investigate Nonenzymatic Histone Glycation. *J. Org. Chem.* **2020**, *85* (3), 1691–1697.

(4) Sibbersen, C.; Palmfeldt, J.; Hansen, J.; Gregersen, N.; Jørgensen, K. A.; Johannsen, M. Development of a Chemical Probe for Identifying Protein Targets of α-Oxoaldehydes. *Chem. Commun.* **2013**, *49* (38), 4012.

(5) Zhang, J.; Lieu, Y. K.; Ali, A. M.; Penson, A.; Reggio, K. S.; Rabadan, R.; Raza, A.; Mukherjee, S.; Manley, J. L. Disease-Associated Mutation in *SRSF2* Misregulates Splicing by Altering RNA-Binding Affinities. *Proc. Natl. Acad. Sci.* **2015**, *112* (34).

(6) Ma, H.-L.; Bizet, M.; Soares Da Costa, C.; Murisier, F.; De Bony, E. J.; Wang, M.-K.; Yoshimi, A.; Lin, K.-T.; Riching, K. M.; Wang, X.; Beckman, J. I.; Arya, S.; Droin, N.; Calonne, E.; Hassabi, B.; Zhang, Q.-Y.; Li, A.; Putmans, P.; Malbec, L.; Hubert, C.; Lan, J.; Mies, F.; Yang, Y.; Solary, E.; Daniels, D. L.; Gupta, Y. K.; Deplus, R.; Abdel-Wahab, O.; Yang, Y.-G.; Fuks, F. SRSF2 Plays an Unexpected Role as Reader of m5C on mRNA, Linking Epitranscriptomics to Cancer. *Mol. Cell* **2023**, *83* (23), 4239-4254.e10.

(7) Sjoblom, N. M.; Kelsey, M. M. G.; Scheck, R. A. A Systematic Study of Selective Protein Glycation. *Angew. Chem. Int. Ed.* **2018**, *57* (49), 16077–16082.

(8) Mulligan, V. K.; Workman, S.; Sun, T.; Rettie, S.; Li, X.; Worrall, L. J.; Craven, T. W.; King, D. T.; Hosseinzadeh, P.; Watkins, A. M.; Renfrew, P. D.; Guffy, S.; Labonte, J. W.; Moretti, R.; Bonneau, R.; Strynadka, N. C. J.; Baker, D. Computationally Designed Peptide Macrocycle Inhibitors of New Delhi Metallo-β-Lactamase 1. *Proc. Natl. Acad. Sci.* **2021**, *118* (12), e2012800118.

(9) Renfrew, P. D.; Choi, E. J.; Bonneau, R.; Kuhlman, B. Incorporation of Noncanonical Amino Acids into Rosetta and Use in Computational Protein-Peptide Interface Design. *PLoS ONE* **2012**, *7* (3), e32637.

(10) Kappel, K.; Jarmoskaite, I.; Vaidyanathan, P. P.; Greenleaf, W. J.; Herschlag, D.; Das, R. Blind Tests of RNA–Protein Binding Affinity Prediction. *Proc. Natl. Acad. Sci.* **2019**, *116* (17), 8336–8341.

(11) Daubner, G. M.; Cléry, A.; Jayne, S.; Stevenin, J.; Allain, F. H.-T. A Syn–Anti Conformational Difference Allows SRSF2 to Recognize Guanines and Cytosines Equally Well. *EMBO J.* **2012**, *31* (1), 162–174.

(12) Gao, Q.; Jacob-Dolan, J. W.; Scheck, R. A. Parkinsonism-Associated Protein DJ-1 Is an Antagonist, Not an Eraser, for Protein Glycation. *Biochemistry* **2023**.
